# BRASSINOSTEROID-SIGNALING KINASE 3, a plasma membrane-associated scaffold protein involved in early brassinosteroid signaling

**DOI:** 10.1371/journal.pgen.1007904

**Published:** 2019-01-07

**Authors:** Hong Ren, Björn C. Willige, Yvon Jaillais, Sa Geng, Mee Yeon Park, William M. Gray, Joanne Chory

**Affiliations:** 1 Howard Hughes Medical Institute, Salk Institute for Biological Studies, La Jolla, California, United States of America; 2 Plant Biology Laboratory, Salk Institute for Biological Studies, La Jolla, California, United States of America; 3 Department of Plant and Microbial Biology, University of Minnesota, Saint Paul, Minnesota, United States of America; 4 Laboratoire Reproduction et Développement des Plantes, Université de Lyon, ENS de Lyon, UCB Lyon 1, CNRS, INRA, Lyon, France; 5 Donald Danforth Plant Science Center, Saint Louis, Missouri, United States of America; The University of North Carolina at Chapel Hill, UNITED STATES

## Abstract

Brassinosteroids (BRs) are steroid hormones essential for plant growth and development. The BR signaling pathway has been studied in some detail, however, the functions of the BRASSINOSTEROID-SIGNALING KINASE (BSK) family proteins in the pathway have remained elusive. Through forward genetics, we identified five semi-dominant mutations in the *BSK3* gene causing *BSK3* loss-of-function and decreased BR responses. We therefore investigated the function of BSK3, a receptor-like cytoplasmic kinase, in BR signaling and plant growth and development. We find that BSK3 is anchored to the plasma membrane via N-myristoylation, which is required for its function in BR signaling. The N-terminal kinase domain is crucial for BSK3 function, and the C-terminal three tandem TPR motifs contribute to BSK3/BSK3 homodimer and BSK3/BSK1 heterodimer formation. Interestingly, the effects of BSK3 on BR responses are dose-dependent, depending on its protein levels. Our genetic studies indicate that kinase dead BSK3^K86R^ protein partially rescues the *bsk3-1* mutant phenotypes. BSK3 directly interacts with the BSK family proteins (BSK3 and BSK1), BRI1 receptor kinase, BSU1 phosphatase, and BIN2 kinase. BIN2 phosphorylation of BSK3 enhances BSK3/BSK3 homodimer and BSK3/BSK1 heterodimer formation, BSK3/BRI1 interaction, and BSK3/BSU1 interaction. Furthermore, we find that BSK3 upregulates *BSU1* transcript and protein levels to activate BR signaling. *BSK3* is broadly expressed and plays an important role in BR-mediated root growth, shoot growth, and organ separation. Together, our findings suggest that BSK3 may function as a scaffold protein to regulate BR signaling. The results of our studies provide new insights into early BR signaling mechanisms.

## Author summary

Steroid hormones exist in both animals and plants. Brassinosteroids (BRs) are steroid hormones that regulate numerous growth and developmental processes throughout the plant life cycle. Discovered in 2008, the BRASSINOSTEROID-SIGNALING KINASE (BSK) protein family is composed of twelve members. However, the functions of these receptor-like cytoplasmic kinases in the BR signaling pathway are poorly understood. Here, we focus on BSK3 and investigate its function in BR signaling and plant growth and development. Our genetic and biochemical studies suggest that BSK3 may function as a scaffold protein to regulate BR signaling. BSK3 is important for BR-mediated root growth, shoot growth, and organ separation. BSK3 activates BR signaling via upregulating *BSU1* transcript levels, and BIN2 phosphorylation of BSK3 influences BSK3 interactions with other signaling components, revealing new layers of regulation in BR signaling. Together, our findings elucidate the function of BSK3 in BR signaling and plant growth and development, and provide new insights into early BR signaling mechanisms.

## Introduction

Brassinosteroids (BRs) are steroid hormones that are essential for plant growth and development. BRs regulate a wide range of cellular, physiological, and developmental processes, including cell expansion, cell division, stem elongation, root development, leaf growth, organ boundary formation, vascular development, male fertility, and stomatal development [1, 2]. BRs are mainly perceived by the plasma membrane-localized leucine-rich repeat (LRR) receptor kinase BRASSINOSTEROID INSENSITIVE 1 (BRI1) [3, 4]. Direct binding of BRs to the extracellular BR-binding domain composed of the island domain and LRR22 of BRI1 [5] causes the release of BRI1 KINASE INHIBITOR 1 (BKI1) from BRI1 to the cytosol [6, 7] and promotes the interaction between BRI1 and BRI1-ASSOCIATED RECEPTOR KINASE 1 (BAK1) [8–10]. Structural studies suggest that BRs act as “molecular glues” to enhance BRI1 and BAK1 association [11, 12]. BRI1 and BAK1 transphosphorylate each other to fully activate BRI1 and initiate a phosphorylation/dephosphorylation-mediated signaling cascade to regulate nuclear gene expression [13]. The activated BRI1 phosphorylates two plasma membrane-associated receptor-like cytoplasmic kinases BRASSINOSTEROID-SIGNALING KINASE 1 (BSK1) and CONSTITUTIVE DIFFERENTIAL GROWTH 1 (CDG1) to activate the phosphatase BRI1 SUPPRESSOR 1 (BSU1) [14–17]. BSU1 subsequently inactivates the GLYCOGEN SYNTHASE KINASE 3 (GSK3)-like kinase BRASSINOSTEROID INSENSITIVE 2 (BIN2) by dephosphorylating BIN2 phosphotyrosine residue pTyr200 [16, 18]. BIN2 and protein phosphatase-2A (PP2A) antagonistically regulate the phosphorylation status of BRASSINAZOLE-RESISTANT 1 (BZR1) and BRI1-EMS-SUPPRESSOR 1 (BES1) transcription factors [19–22]. Upon inhibition of BIN2 by BSU1 [16] and SCF^KIB1^-mediated BIN2 degradation [23], dephosphorylated BZR1 and BES1 accumulate in the nucleus to regulate the expression of hundreds of genes [20, 24–28]. These BR-responsive genes then control various cellular, physiological, and developmental processes.

The BR signaling pathway has been studied in some detail, however, the functions of the BSK family proteins in the pathway have remained elusive. The founding members of this family are BSK1 and BSK2, which were identified as BR-responsive proteins by two-dimensional difference gel electrophoresis and mass spectrometry [15]. The BSK protein family is composed of twelve members, which contain an N-terminal kinase domain and C-terminal tetratricopeptide repeat (TPR) motifs [15]. Although called BRASSINOSTEROID-SIGNALING KINASES, the available results indicate that not all members are involved in BR signaling. SHORT SUSPENSOR (SSP/BSK12) activates the YODA mitogen-activated protein kinase pathway to regulate suspensor development during embryogenesis [29], which has not been shown to be regulated by BRs. Biochemical and genetic studies have implicated numerous BSK members (BSK1, 2, 3, 4, 5, 6, 8, and 11) as positive regulators in BR signaling [15, 30]. BRI1 phosphorylation of BSK1 at Ser230 promotes BSK1 interaction with BSU1 phosphatase [15, 16], however, the mechanism underlying BSK1-mediated BSU1 activation is not known [17]. *bsk3/4/6/7/8* pentuple mutants exhibit multiple growth defects, including reduced rosette size, leaf curling, and enhanced leaf inclination [30]. However, these mutants are phenotypically very different from BR deficient and response mutants, which exhibit severe growth defects, including dark green and rounded leaves, reduced male fertility, and reduced silique growth [3, 31–33]. Together, the functional importance of the BSK family members in BR signaling and BR-mediated plant growth and development remains unclear.

To identify novel regulators in BR signaling and responses, we took a forward genetic approach to screen for mutations that cause decreased BR responses in Arabidopsis. Our genetic screen identified five semi-dominant mutations causing decreased BR responses. Through positional cloning, we determined that the mutations occurred in the *BSK3* gene (At4g00710), which encodes a receptor-like cytoplasmic kinase. Our genetic, molecular, and biochemical studies demonstrate that BSK3 plays an important role in BR signaling and BR-mediated plant growth and development.

## Results

### A root-based genetic screen identifies five semi-dominant mutations in *BSK3* causing decreased BR responses

**Fig 1:**
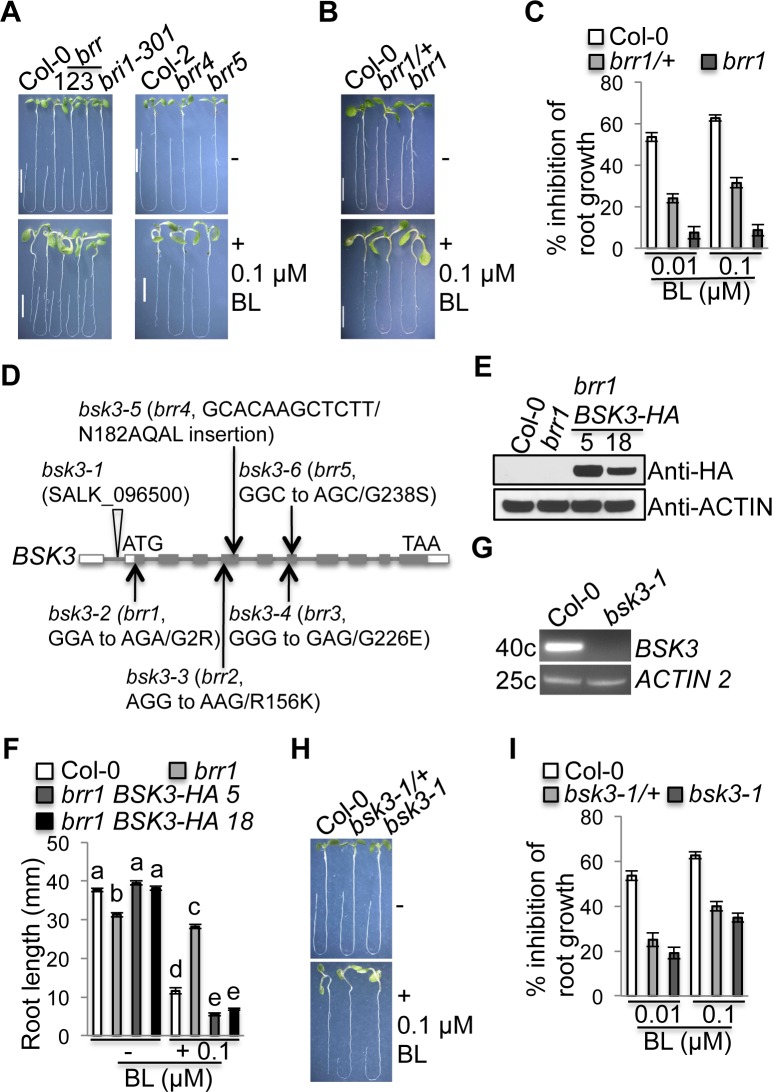
Identification of five semi-dominant mutations in *BSK3*. **(**A-C) Root growth of Col-0 and *brr* mutants (+/- BL). (D) The gene structure of *BSK3*. Arrows point to the positions of five identified *BSK3* mutations. White boxes represent 5’ UTR and 3’ UTR. Gray boxes and lines represent exons and introns, respectively. Triangle represents the T-DNA insert, and the insertion is in the first intron in the 5’ UTR of *BSK3*. (E) Western blot analysis of BSK3-HA protein expression in two *brr1 BSK3-HA* lines. Thirty micrograms of total proteins were loaded. (F) Primary root length of Col-0, *brr1*, and *brr1 BSK3-HA* seedlings. Different letters above the bars indicate significant differences (P < 0.05). (G) RT-PCR analysis of *BSK3* gene expression. ACTIN2-FW/ACTIN2-RV and BSK3-FW/BSK3-RV primers were used to amplify *ACTIN2* (25 cycles) and *BSK3* (40 cycles) transcripts, respectively. (H and I) Root growth of Col-0, *bsk3-1/+*, and *bsk3-1* seedlings (+/- BL). (A-C and E-I) Seedlings were grown in the light for 7 days. Error bars represent SEM (n = 36–60). Scale bar = 0.5 cm.

Previous genetic screens for mutations that cause decreased BR responses in Arabidopsis only successfully identified two BR signaling regulators, BRI1 and BIN2 [3, 18, 31, 34, 35]. The identified *bri1* and *bin2* mutants are dwarf or semi-dwarf plants that exhibit pleiotropic growth defects, including reduced rosette size, dark green and rounded leaves, and reduced male fertility. We reasoned that previous screens missed mutants that do not exhibit *bri1*/*bin2*-like shoot phenotypes. To identify mutants affecting novel loci involved in BR signaling, we carried out a root-based genetic screen for mutations that cause decreased BR responses in Arabidopsis. Wild-type Columbia (Col) seedlings exhibited reduced root growth when grown on 0.1 μM brassinolide (BL, the most active BR) (S1A Fig). We screened ethyl methanesulfonate (EMS)-mutagenized (in Col-0) and activation-tagged (in Col-2) populations. Seedlings on 0.1 μM BL exhibiting longer roots were selected. We named these plants *brassinosteroid resistant* (*brr*) mutants. Here, we report five *brr* mutants (*brr1*, *brr2*, and *brr3* in Col-0; *brr4* and *brr5* in Col-2) (Fig 1A). To determine the recessive or dominant nature of these mutations, we crossed all *brr* mutants into the wild-type Col-0 or Col-2 plants. Surprisingly, both heterozygous and homozygous mutants were resistant to 0.01 and 0.1 μM BL. In addition, homozygous mutants had stronger BL resistance (Fig 1B and 1C and S1B–S1E Fig), indicating that all five *brr* mutations are semi-dominant. Although *brr4* and *brr5* were identified from an activation-tagged population, co-segregation analyses of BL and Basta resistance suggested that the mutations were not caused by T-DNA inserts.

We used positional cloning to identify the affected gene of the *brr1* mutant, which exhibited slightly reduced root growth in the absence of BL (Fig 1F). This mutant is caused by a mutation in the *BSK3* gene (At4g00710), which encodes a receptor-like cytoplasmic kinase [15]. The second glycine of the protein was mutated to arginine (Fig 1D). To confirm that *BSK3* is responsible for the mutant phenotypes, we expressed an HA-tagged *BSK3* genomic DNA under the control of the native *BSK3* promoter in the *brr1* mutant. Two independent *brr1* transgenic lines expressing BSK3-HA protein were selected (Fig 1E). Light-grown *brr1 BSK3-HA* seedlings exhibited wild-type-like root length and hypersensitivity to 0.1 μM BL (Fig 1F). These results demonstrate that *BSK3* genomic DNA complements the *brr1* mutant phenotypes, including the root growth defect and BL resistance, confirming that the BSK3^G2R^ mutation confers the *brr1* mutant phenotypes. Subsequent sequencing of the *BSK3* gene in other four *brr* mutants (*brr2* to *brr 5*) revealed that all these mutants are caused by mutations in the *BSK3* gene (Fig 1D). We therefore changed the names of five *brr* mutants to *bsk3-2* (*brr1*), *bsk3-3* (*brr2*), *bsk3-4* (*brr3*), *bsk3-5* (*brr4*), and *bsk3-6* (*brr5*) (Fig 1D).

Since all five identified *bsk3* mutations are semi-dominant mutations causing *BSK3* loss-of-function, we were curious whether the known *bsk3-1* T-DNA insertion mutant (SALK_096500) [15, 30] is also semi-dominant. The T-DNA insertion of *bsk3-1* is located in the first intron in the 5’ UTR of *BSK3* (Fig 1D). RT-PCR did not detect any *BSK3* transcripts in *bsk3-1* (Fig 1G), indicating that *bsk3-1* is probably a null mutant. We crossed the *bsk3-1* mutant into the wild-type Col-0 plants. Both heterozygous and homozygous mutant seedlings exhibited resistance to 0.01 and 0.1 μM BL. In addition, homozygous mutant seedlings exhibited stronger BL resistance (Fig 1H and 1I). These results suggest that the *bsk3-1* mutant is also semi-dominant, indicating a dose-dependent effect of BSK3 on BR responses.

### BSK3 is an N-myristoylated protein that localizes to the plasma membrane

**Fig 2:**
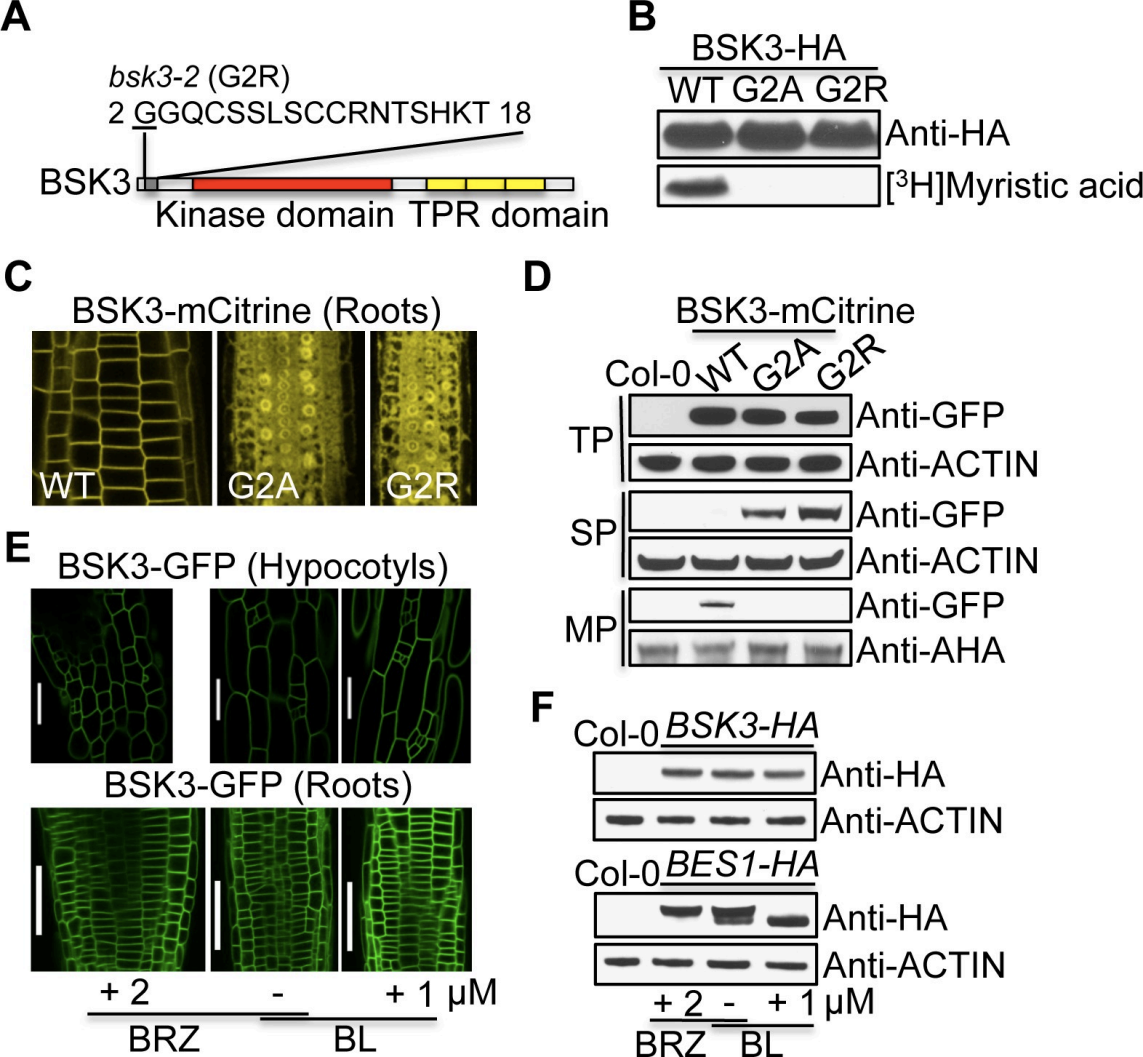
BSK3 is an N-myristoylated protein that localizes to the plasma membrane. (A) A putative BSK3 N-myristoylation sequence GGQCSSLSCCRNTSHKT predicted by NMT, the MYR predictor. (B) *In vitro* myristoylation assays. (C) Subcellular localization of BSK3^WT/G2A/G2R^-mCitrine proteins. Root tip regions of 4-day-old light-grown seedlings were analyzed. (D) Western blots detecting BSK3^WT/G2A/G2R^-mCitrine proteins. Proteins were extracted from 7-day-old light-grown seedlings. Twenty to thirty micrograms of total proteins (TP), soluable proteins (SP), and microsomal proteins (MP) were loaded. AHA (Arabidopsis H^+^-ATPases) proteins were detected by an anti-AHA antibody. (E) Subcellular localization of BSK3-GFP protein. Seedlings were grown on 2 μM BRZ in the light for 4 days, or 4-day-old light-grown seedlings were treated with 1 μM BL for 2 hours. Scale bars = 50 μm. (F) Western blots detecting BSK3-HA and BES1-HA proteins. Seedlings were grown on 2 μM BRZ for 6 days, or 6-day-old light-grown seedlings were treated with 1 μM BL for 2 hours. Thirty micrograms of total proteins were loaded.

N-myristoylation is a co-translational lipid modification. The mechanisms underlying N-myristoylation are conserved among eukaryotes. Myristic acid is linked to a protein’s N-terminal glycine residue via an amide bond, which is catalyzed by an N-myristoyltransferase [36–38]. Analysis of BSK3 protein sequence using NMT-The MYR Predictor (http://mendel.imp.ac.at/myristate/SUPLpredictor.htm) indicated that BSK3 has a putative myristoylation sequence at its N-terminus (Fig 2A), suggesting that it may be an N-myristoylated protein. To determine whether BSK3 is an N-myristoylated protein, we performed *in vitro* myristoylation assays. The TNT SP6 high-yield wheat germ protein expression system was used to synthesize HA-tagged BSK3 protein in the presence of [^3^H]myristic acid, and reaction products were analyzed by SDS-PAGE, western blot, and fluorography. Wheat germ extracts contain the N-myristoyltransferase, which can modify proteins. Consistent with the prediction by NMT-The MYR Predictor, wild-type BSK3-HA was labeled (Fig 2B). The second glycine of BSK3, a putative myristoylation site, was mutated to arginine in the *bsk3-2* mutant (Fig 2A). To determine whether this G2R mutation affects BSK3 myristoylation, we performed PCR-based site-directed mutagenesis to create BSK3^G2R^ mutation and examined the myristoylation of this mutant protein. It was previously reported that the G2A mutation blocks the myristoylation of N-myristoylated proteins [39]. As a control, we included BSK3^G2A^-HA protein in the assays. Unlike the wild-type protein, BSK3^G2A^-HA and BSK3^G2R^-HA mutant proteins were not modified by [^3^H]Myristic acid (Fig 2B), indicating that these two mutations block BSK3 myristoylation.

N-myristoylation confers a tendency to associate with membranes [37], indicating that BSK3 may be a membrane-associated protein. To examine BSK3 subcellular localization, we generated transgenic Arabidopsis plants expressing *BSK3-mCitrine* driven by the native *BSK3* promoter. BSK3-mCitrine protein localized exclusively to the plasma membrane (Fig 2C). To determine whether N-myristoylation is required for BSK3 plasma membrane localization, we examined the subcellular localization of BSK3^G2A^-mCitrine and BSK3^G2R^-mCitrine proteins. Unlike the wild-type protein, these two mutant proteins were mislocalized in the nucleus and cytosol (Fig 2C). To further show that BSK3-mCitrine is a plasma membrane-localized protein while BSK3^G2A^-mCitrine and BSK3^G2R^-mCitrine mutant proteins are not, we performed subcellular protein fractionation. Consistent with our confocal observations, BSK3-mCitrine protein was only detected in the membrane fraction (Fig 2D). By contrast, BSK3^G2A^-mCitrine and BSK3^G2R^-mCitrine mutant proteins were only detected in the soluble fraction (Fig 2D). These results indicate that N-myristoylation is required for BSK3 plasma membrane localization.

To determine whether BRs regulate BSK3 subcellular localization, we treated light-grown *BSK3pro*:*BSK3-GFP* seedlings with 2 μM brassinazole (BRZ, a BR biosynthesis inhibitor) for four days and 1 μM BL for two hours, respectively. Seedlings treated with BRZ and BL showed BSK3-GFP localization exclusively on the plasma membrane in the hypocotyls and roots (Fig 2E), indicating that BRs do not regulate BSK3 plasma membrane localization. Since the effects of BSK3 on BR responses are dose-dependent, depending on BSK3 protein levels (Fig 1H and 1I), we wondered whether BSK3 protein levels are regulated by BRs. Light-grown *BSK3pro*:*BSK3-HA* and *BES1pro*:*BES1-HA* seedlings were treated with 2 μM BRZ for six days and 1 μM BL for two hours, respectively. BRZ and BL treatments caused increased and reduced BES1-HA protein phosphorylation, indicating decreased and increased BR responses, respectively (Fig 2F). However, BSK3-HA protein levels were not altered by BRZ and BL treatments (Fig 2F), suggesting that BRs do not regulate BSK3 protein levels.

### Kinase dead BSK3^K86R^ protein partially rescues the *bsk3-1* mutant phenotypes

All BSK family proteins contain an N-terminal kinase domain, however, contradictory results have been reported regarding whether these proteins have kinase activity *in vitro*. Autophosphorylation of BSK1, 3, 5, 6, 8, and 11 were not detected in kinase assay reactions containing Mg^2+^ or Mn^2+^ [15, 30, 40]. However, BSK1 was shown to exhibit kinase activity, requiring Mn^2+^ as a divalent cation cofactor [41, 42]. A luciferase-based kinase assay using affinity-purified BSK8-GFP protein from Arabidopsis seedlings showed that BSK8 has kinase activity [43]. Caution should be taken, however, regarding the detected BSK8 kinase activity, as kinases copurifying with BSK8-GFP may contribute to the detected kinase activity. OsBSK3, a BSK3 ortholog in rice, was shown to exhibit weak autophosphorylation activity in kinase assay reactions containing Mg^2+^ or Mn^2+^. However, a kinase dead OsBSK3 protein was not included as a negative control. Furthermore, the authors could not confidently claim that OsBSK3 is an active kinase since the autophosphorylation signals were too weak [44]. Together, all these results suggest that further research is needed to conclude whether the BSK family proteins are real kinases.

**Fig 3:**
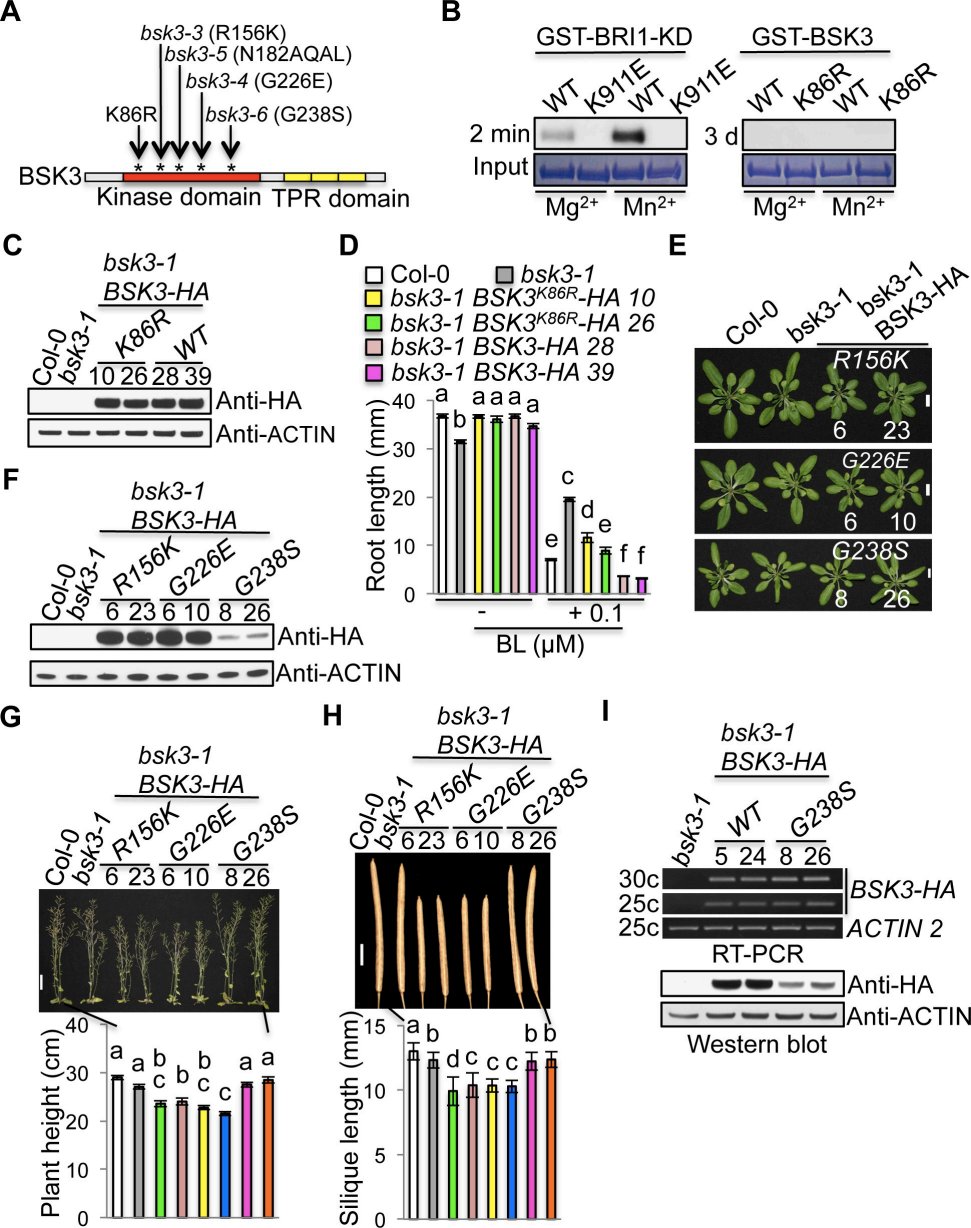
Kinase dead BSK3^K86R^ protein partially rescues the *bsk3-1* mutant phenotypes. (A) BSK3 mutations in the kinase domain. The kinase domain (58–312 aa) of BSK3 was predicted by SMART (Simple Modular Architecture Research Tool, http://smart.embl-heidelberg.de/). (B) *In vitro* kinase assays. About 10–20 μg of GST fusion proteins were loaded for kinase assays. Dried protein gels were exposed to films for 2 minutes (GST-BRI1-KD and GST-BRI1-KD^K911E^) or 3 days (GST-BSK3 and GST-BSK3^K86R^) at—80 ^o^C. (C) Western blot analyses of BSK3-HA and BSK3^K86R^-HA protein expression. (D) Primary root length of 7-day-old light-grown seedlings. Seedlings were grown on ½ LS agar plates with or without 0.1 μM BL for 7 days. Error bars represent SEM (n = 40–50). (E) Four-week-old plants. Primary bolts were removed to better observe rosette leaves. Scale bar = 1 cm. (F) Western blot analyses of BSK3^R156K/G226E/G238S^-HA protein expression. (G) Seven-week-old plants. Error bars represent SEM (n = 14–19). Scale bar = 4 cm. (H) Mature siliques. Siliques from 14 to 19 plants (10 siliques per plant) were analyzed. Error bars represent STD (n = 140–190). (I) Semi-quantitative RT-PCR and western blot analyses of *BSK3-HA* and *BSK3*^*G238S*^*-HA* transcript and protein levels, respectively. Homozygous T4 transgenic plants were analyzed. (C, F, and I) Thirty micrograms of total proteins from 7-day-old light-grown seedlings were loaded. (D, G, and H) Different letters above the bars indicate significant differences (P < 0.05).

To determine whether BSK3 has kinase activity, we performed kinase assays to examine BSK3 autophosphorylation. Kinase active GST-BRI1-KD and kinase dead GST-BRI1-KD^K911E^ proteins were included as controls. Like BRI1 receptor kinase, all BSK family proteins contain the highly conserved lysine (K) residue required for binding ATP and the catalytic activity (S2 Fig). We performed PCR-based site-directed mutagenesis to create kinase dead BSK3^K86R^ mutation (Fig 3A and S2 Fig). GST-BRI1-KD protein exhibited kinase activity with an exposure time of two minutes, showing stronger autophosphorylation in the presence of Mn^2+^ (Fig 3B). However, GST-BSK3 protein did not exhibit any autophosphorylation in the presence of either Mg^2+^ or Mn^2+^ despite an exposure time of three days (Fig 3B). These results indicate that unlike BRI1 receptor kinase, BSK3 does not have kinase activity under our conditions.

A previous study reported that kinase dead SSP/BSK12^K78R^ protein could fully complement the short suspensor phenotype of the *ssp* mutants, suggesting that SSP kinase activity may not be required for SSP function in suspensor development during embryogenesis [29]. We used the same strategy to determine whether BSK3 kinase activity, if it has kinase activity *in planta*, is required for BSK3 function in BR signaling. We selected two independent *bsk3-1* transgenic lines expressing *BSK3-HA* or *BSK3*^*K86R*^*-HA* driven by the native *BSK3* promoter, which exhibited similar protein levels (Fig 3C). Like BSK3-HA, kinase dead BSK3^K86R^-HA protein fully complemented the short root phenotype of the *bsk3-1* mutant (Fig 3D). Unexpectedly, *bsk3-1 BSK3-HA* seedlings exhibited hypersensitivity to 0.1 μM BL in the root elongation assays, while *bsk3-1 BSK3*^*K86R*^*-HA* seedlings still exhibited resistance to 0.1 μM BL, although considerably weaker than *bsk3-1* seedlings (Fig 3D). These results indicate that kinase dead BSK3^K86R^-HA protein only partially rescues the *bsk3-1* mutant phenotypes, suggesting that BSK3 kinase activity, or at least BSK3 binding to ATP, may be required for full BSK3 function in BR signaling. As described later, BSK3 may function as a scaffold protein in BR signaling, we could not rule out that the BSK3^K86R^ mutation may affect the scaffold function and thereby impairs BSK3 function in BR signaling.

Our genetic screen identified four mutations (R156K, N182AQAL insertion, G226E, and G238S) in the kinase domain of BSK3 that confer decreased BR responses, suggesting that the kinase domain is crucial for BSK3 function in BR signaling (Figs 1D and 3A). To assess how three missense mutations (R156K, G226E, and G238S) affect BSK3 function, we used the native *BSK3* promoter to express BSK3^R156K^-HA, BSK3^G226E^-HA, and BSK3^G238S^-HA proteins in the *bsk3-1* mutant, and analyzed the ability of these mutant proteins to complement the *bsk3-1* mutant. As described previously, expression of wild-type BSK3-HA protein under the control of the native *BSK3* promoter complemented the *bsk3-1* mutant (Fig 3D). *bsk3-1* plants did not exhibit obvious shoot growth defects. However, *bsk3-1* transgenic plants expressing *BSK3*^*R156K*^*-HA* or *BSK3*^*G226E*^*-HA*, but not *BSK3*^*G238S*^*-HA*, exhibited slightly smaller rosette leaves (Fig 3E). Western blot analyses showed that *bsk3-1 BSK3*^*R156K*^*-HA* and *bsk3-1 BSK3*^*G226E*^*-HA* plants exhibited high levels of protein expression, while *bsk3-1 BSK3*^*G238S*^*-HA* plants exhibited low levels of protein expression (Fig 3F). Consistent with the slight growth defect of rosette leaves, mature *bsk3-1 BSK3*^*R156K*^*-HA* and *bsk3-1 BSK3*^*G226E*^*-HA* plants were shorter than *bsk3-1* plants (Fig 3G) and had smaller siliques (Fig 3H), indicating defective shoot and silique growth. The above results suggest that BSK3^R156K^ and BSK3^G226E^ mutations may have dominant-negative effects, interfering with the function of additional BSK members in BR signaling.

We noticed that *bsk3-1 BSK3*^*G238S*^*-HA* plants expressed low levels of BSK3^G238S^-HA protein. Twenty-six independent transgenic lines were screened, and Lines 8 and 26 exhibited highest protein expression. However, BSK3^G238S^-HA protein levels were still much lower than those of BSK3^R156K^-HA and BSK3^G226E^-HA proteins (Fig 3F), suggesting that the G238S mutation may affect BSK3 protein stability. To test this hypothesis, we analyzed *bsk3-1* transgenic plants expressing *BSK3-HA* or *BSK3*^*G238S*^*-HA* under the control of the native *BSK3* promoter. Semi-quantitative RT-PCR showed that *bsk3-1 BSK3-HA* lines 5 and 24 exhibited similar levels of transcripts as those of *bsk3-1 BSK3*^*G238S*^*-HA* lines 8 and 26 (Fig 3I). However, western blot analyses showed that BSK3^G238S^-HA protein levels were much lower than those of BSK3-HA protein (Fig 3I). Together, these results suggest that the G238S mutation may indeed reduce BSK3 protein stability.

### BSK3 physically interacts with BRI1 receptor kinase, BSU1 phosphatase, BIN2 kinase, and the BSK family members

**Fig 4:**
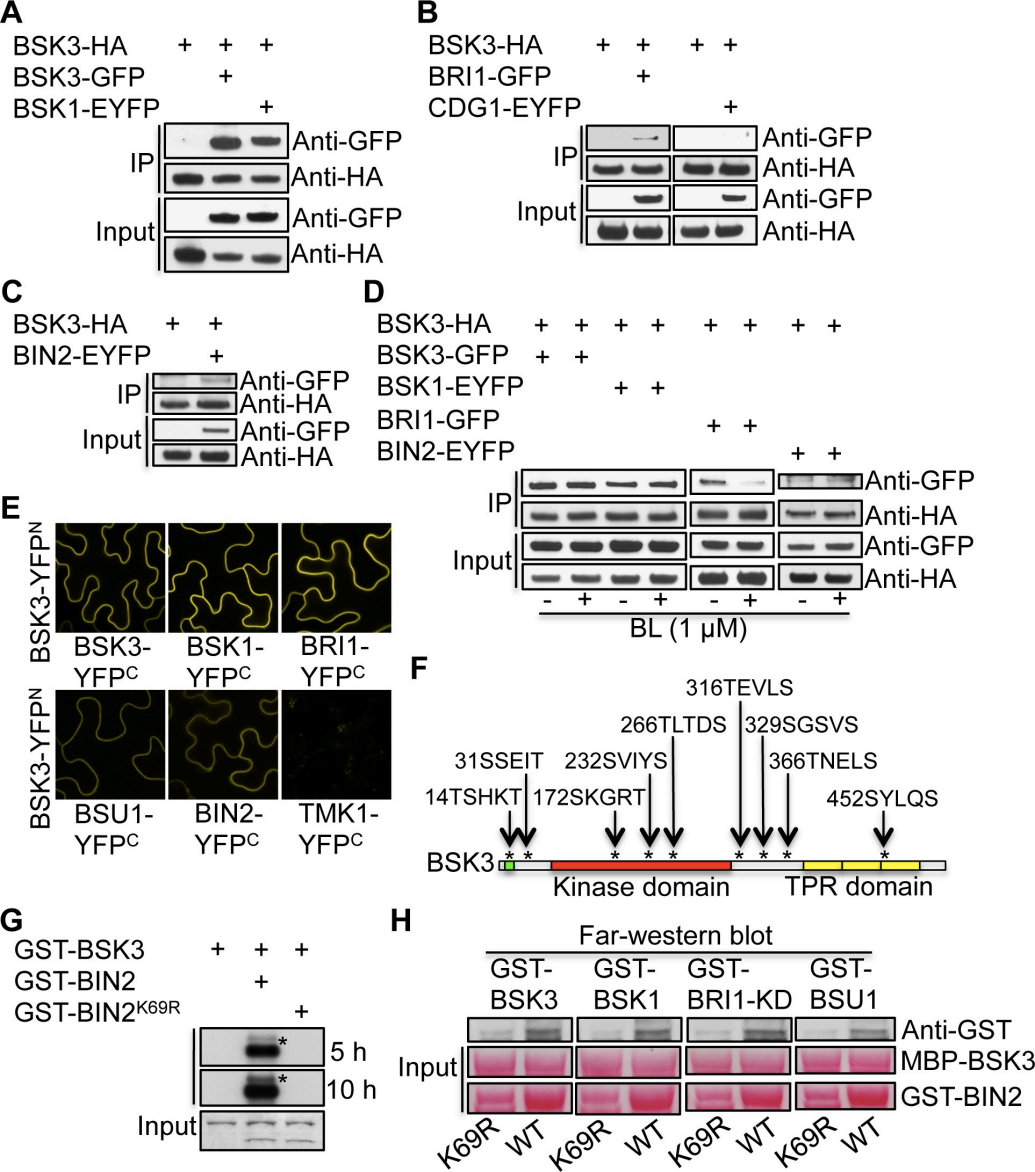
BSK3 interacts with BSK3, BSK1, BRI1, BSU1, and BIN2. **(**A-C) Co-IP assays detecting BSK3-HA interactions with BSK3-GFP, BSK1-EYFP, BRI1-GFP, and BIN2-EYFP. (D) Effects of BL on protein interactions in Arabidopsis. Seedlings were treated with 1 μM BL for 30 minutes. (E) BiFC assays in *Nicotiana benthamiana* leaves. (F) Putative BIN2 phosphorylation sites of BSK3. (G) *In vitro* kinase assays. Asterisks (*) indicate the phosphorylated BSK3. The band below the phosphorylated BSK3 is the autophosphorylated BIN2. Dried protein gels were exposed to films for 5 or 10 hours at—80 ^o^C. (H) Far-western blot analyses of BSK3 interactions with BSK3, BSK1, BRI1, and BSU1. MBP-BSK3-bound GST-BSK1, GST-BSK3, GST-BRI1-KD, and GST-BSU1 proteins were detected by an anti-GST antibody. Nitrocellulose membranes were stained with ponceau S to detect MBP-BSK3 (97.5 kDa), GST-BIN2 (70 kDa), and GST-BIN2^K69R^ (70 kDa) proteins. (A-D) Total 300–870 μg of microsomal proteins from 7 or 8-day-old light-grown seedlings were immunoprecipitated using an anti-HA antibody.

BSK3 was previously shown to be a substrate of BRI1 kinase *in vitro* [15], however, the precise function of BSK3 in BR signaling is not known. To understand the role of BSK3 in BR signaling, we sought to identify BSK3 interactors by testing the interactions between BSK3 and the known BR signaling regulators: BRI1 receptor kinase, CDG1 kinase, BSU1 phosphatase, and BIN2 kinase. To know whether BSK3 can form a homodimer or heterodimer with the BSK family members, we also tested BSK3/BSK3 and BSK3/BSK1 protein interactions. We generated transgenic Arabidopsis plants co-expressing BSK3-HA and BRI1-GFP, CDG1-EYFP, BSU1-EYFP, BIN2-EYFP, BSK3-GFP, or BSK1-EYFP proteins. We immunoprecipitated BSK3-HA protein using an anti-HA antibody and detected the co-immunoprecipitated (co-IPed) GFP/EYFP fusion proteins by western blots using an anti-GFP antibody. Since a non-specific protein recognized by the anti-GFP antibody and the antibody heavy chain prevented us detecting BSU1-EYFP and BSK3-HA proteins, respectively, we could not successfully determine BSK3-HA and BSU1-EYFP protein interaction by co-IP assays. BSK3-HA co-IPed with BSK3-GFP (Fig 4A), BSK1-EYFP (Fig 4A), BRI1-GFP (Fig 4B), BIN2-EYFP (Fig 4C), but not CDG1-EYFP (Fig 4B). We further confirmed these protein interactions by GST pull-down and bimolecular fluorescence complementation (BiFC) assays, and BSU1 was included in these assays. GST-BSK3 protein pulled down BSK3-HA and BSK1-FLAG, and GST-BRI1-KD, GST-BSU1, and GST-BIN2 proteins pulled down BSK3-HA (S3 Fig). Plasma membrane-localized yellow fluorescent signals were observed in the leaf epidermal cells when BSK3-YFP^N^ were transiently co-expressed with BSK3-YFP^C^, BSK1-YFP^C^, BRI1-YFP^C^, BSU1-YFP^C^, or BIN2-YFP^C^, but not with the plasma membrane-localized receptor kinase TMK1-YFP^C^ [45], in *Nicotiana benthamiana* leaves (Fig 4E). The expression of these proteins was confirmed by western blots using anti-Myc and anti-HA antibodies (S4 Fig). Together, our co-IP, GST pull-down, and BiFC results demonstrate that BSK3 directly interacts with BSK3, BSK1, BRI1, BSU1, and BIN2 in the BR signaling pathway.

To know whether BRs regulate BSK3 interactions with BSK3, BSK1, BRI1, and BIN2, we treated transgenic Arabidopsis seedlings co-expressing BSK3-HA and BSK3-GFP, BSK1-EYFP, BRI1-GFP, or BIN2-EYFP proteins with 1 μM BL for 30 minutes and performed co-IP assays. Consistent with previous findings [15], BR treatment reduced BSK3-HA and BRI1-GFP protein interaction (Fig 4D). However, the interactions between BSK3-HA and BSK3-GFP, BSK1-EYFP, or BIN2-EYFP proteins were not affected by BR treatment (Fig 4D). These results indicate that BRs do not regulate BSK3 and BIN2 protein interaction and the formation of BSK3/BSK3 homodimers and BSK3/BSK1 heterodimers.

Our co-IP, GST pull-down, and BiFC assays demonstrated that BSK3 physically interacts with the GSK3-like kinase BIN2 at the plasma membrane (Fig 4 and S3 Fig). We were curious whether BSK3 is a substrate of BIN2 kinase, and therefore searched for the GSK3 consensus phosphorylation motifs (S/T-X-X-X-S/T, X is any amino acid) in BSK3. BSK3 contains nine putative GSK3 consensus phosphorylation motifs (Fig 4F), suggesting that BIN2 may be able to phosphorylate BSK3. To determine whether BSK3 is a BIN2 kinase substrate, we performed kinase assays using GST-BSK3, kinase active GST-BIN2, and kinase dead GST-BIN2^K69R^ proteins. A previous study showed that GST protein is not phosphorylated by BIN2 kinase, suggesting that it is not a BIN2 kinase substrate [21]. BSK3 phosphorylation was observed when GST-BSK3 was incubated with GST-BIN2, but not with GST-BIN2^K69R^ (Fig 4G), indicating that BIN2 kinase phosphorylates BSK3. Our results are consistent with the previous finding that BIN2 kinase phosphorylates BSK3 *in vitro* [30]. Together, these findings suggest that BSK3 is a substrate of BIN2 kinase.

To understand the biological significance of BSK3 phosphorylation by BIN2 in BR signaling, we performed *in vitro* kinase assays and far-western blot assays to examine whether BIN2 phosphorylation of BSK3 may affect BSK3 interactions with BSK3, BSK1, BRI1, and BSU1. MBP-BSK3, GST-BIN2, and GST-BIN2^K69R^ proteins expressed in *E*. *coli* cells were purified, and the expression levels of GST-BIN2^K69R^ were lower than those of GST-BIN2. Equal amounts of MBP-BSK3 proteins were incubated with GST-BIN2 or GST-BIN2^K69R^ for kinase assays. After kinase assays, MBP-BSK3 (97.5 kDa) and GST-BIN2 or GST-BIN2^K69R^ (70 kDa) proteins were separated by SDS-PAGE and transferred onto nitrocellulose membranes. GST-BSK3, GST-BSK1, GST-BRI1-KD, and GST-BSU1 proteins synthesized using the TNT SP6 high-yield wheat germ protein expression system were incubated with nitrocellulose membrane strips containing only MBP-BSK3 without GST-BIN2 or GST-BIN2^K69R^. We detected MBP-BSK3-bound GST fusion proteins using an anti-GST antibody. Interestingly, BIN2 phosphorylation of BSK3 enhanced MBP-BSK3 interactions with GST-BSK3, GST-BSK1, GST-BRI1-KD, and GST-BSU1 (Fig 4H). These results suggest that BSK3 phosphorylation by BIN2 promotes BSK3/BSK3 homodimer and BSK3/BSK1 heterodimer formation, BSK3/BRI1 interaction, and BSK3/BSU1 interaction.

### TPR motifs are not essential, but are required for the full function of BSK3 in BR signaling

**Fig 5:**
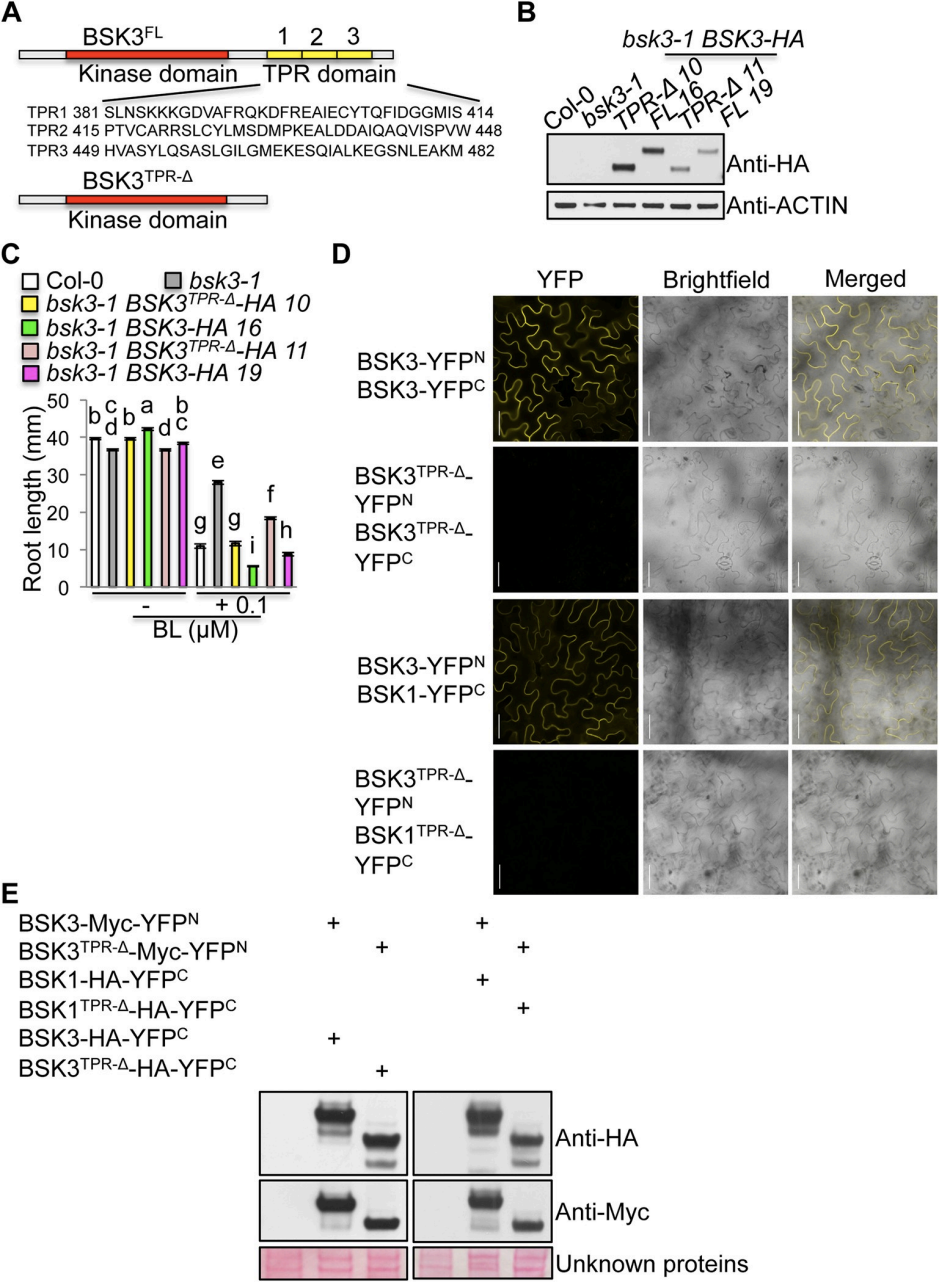
TPR deletion BSK3 protein fully rescues the *bsk3-1* mutant phenotypes. (A) The structures of full-length (FL) and TPR deletion (TPR-Δ) BSK3 proteins. BSK3 has three tandem TPR motifs predicted by TPRpred. (B) Western blot analyses of BSK3-HA and BSK3^TPR-Δ^-HA protein expression. Twenty-five micrograms of total proteins from 7-day-old light-grown seedlings were loaded. (C) Primary root length of 7-day-old light-grown seedlings grown on ½ LS agar plates with or without 0.1 μM BL. Error bars represent SEM (n = 45–67). Different letters above the bars indicate significant differences (P < 0.05). (D) BiFC assays in *Nicotiana benthamiana* leaves. Scale bar = 100 μm. (E) Western blot analyses of transiently expressed proteins in *Nicotiana benthamiana* leaves. Twenty-five micrograms of microsomal proteins were loaded. Unknown proteins stained with Ponceau S are shown as loading controls.

Analysis of BSK3 protein sequence using TPRpred (http://toolkit.tuebingen.mpg.de/tprpred) indicated that BSK3 has three C-terminal tandem TPR motifs (Fig 5A). Our genetic screen identified five mutations in BSK3, surprisingly, no mutations were identified in the TPR motifs. We were curious whether the TPR motifs are essential for BSK3 function in BR signaling. We therefore performed PCR-based site-directed mutagenesis to create a truncated BSK3 protein without the C-terminal three tandem TPR motifs (BSK3^TPR-Δ^) (Fig 5A), and examined whether this mutant protein could rescue the *bsk3-1* mutant phenotypes, including the slight root growth defect and BR resistance.

Two independent *bsk3-1* transgenic lines expressing *BSK3-HA* or *BSK3*^*TPR-Δ*^*-HA* driven by the native *BSK3* promoter were selected. *bsk3-1 BSK3-HA* line 16 and *bsk3-1 BSK3*^*TPR-Δ*^*-HA* line 10 expressed similar high protein levels, while *bsk3-1 BSK3-HA* line 19 and *bsk3-1 BSK3*^*TPR-Δ*^*-HA* line 11 expressed similar low protein levels (Fig 5B). Like the wild-type seedlings, 0.1 μM BL-treated *bsk3-1 BSK3-HA* and *bsk3-1 BSK3*^*TPR-Δ*^*-HA* seedlings exhibited shorter roots, indicating loss of *bsk3-1*’s BR resistance phenotype (Fig 5C). Interestingly, *bsk3-1 BSK3*^*TPR-Δ*^*-HA* seedlings exhibited less sensitivity to 0.1 μM BL than *bsk3-1 BSK3-HA* seedlings (Fig 5C). Unlike line 11, *bsk3-1 BSK3*^*TPR-Δ*^*-HA* line 10 seedlings expressed higher protein levels and fully rescued the root growth defect and BR resistance phenotype of *bsk3-1* seedlings (Fig 5C). These results demonstrate that BSK3^TPR-Δ^-HA protein fully complements the *bsk3-1* mutant phenotypes, indicating that the C-terminal three tandem TPR motifs are not essential for BSK3 function in BR signaling. Interestingly, expressed at similar levels from the native *BSK3* promoter, BSK3^TPR-Δ^-HA protein was less efficient to rescue the root growth defect and BR resistance phenotype of *bsk3-1* seedlings than BSK3-HA protein (Fig 5C). These results indicate that TPR deletion impairs BSK3 function, suggesting that TPR motifs are required for the full function of BSK3 in BR signaling.

Previously, we demonstrated that BSK3 interacts with the BSK family members (BSK1 and BSK3), BRI1, BSU1, and BIN2. We were curious whether TPR motifs are involved in these protein interactions. We therefore performed BiFC assays to test BSK3^TPR-Δ^ interactions with BSK1^TPR-Δ^, BSK3^TPR-Δ^, BRI1, BSU1, and BIN2. BSK3^TPR-Δ^ does not interact with BSK1^TPR-Δ^ and BSK3^TPR-Δ^ (Fig 5D and 5E), while BSK3^TPR-Δ^ still interacts with BRI1, BSU1, and BIN2 (S5 Fig). These results indicate that TPR deletion impairs BSK3/BSK3 homodimer and BSK3/BSK1 heterodimer formation.

### *BSK3* is broadly expressed throughout plant development

**Fig 6:**
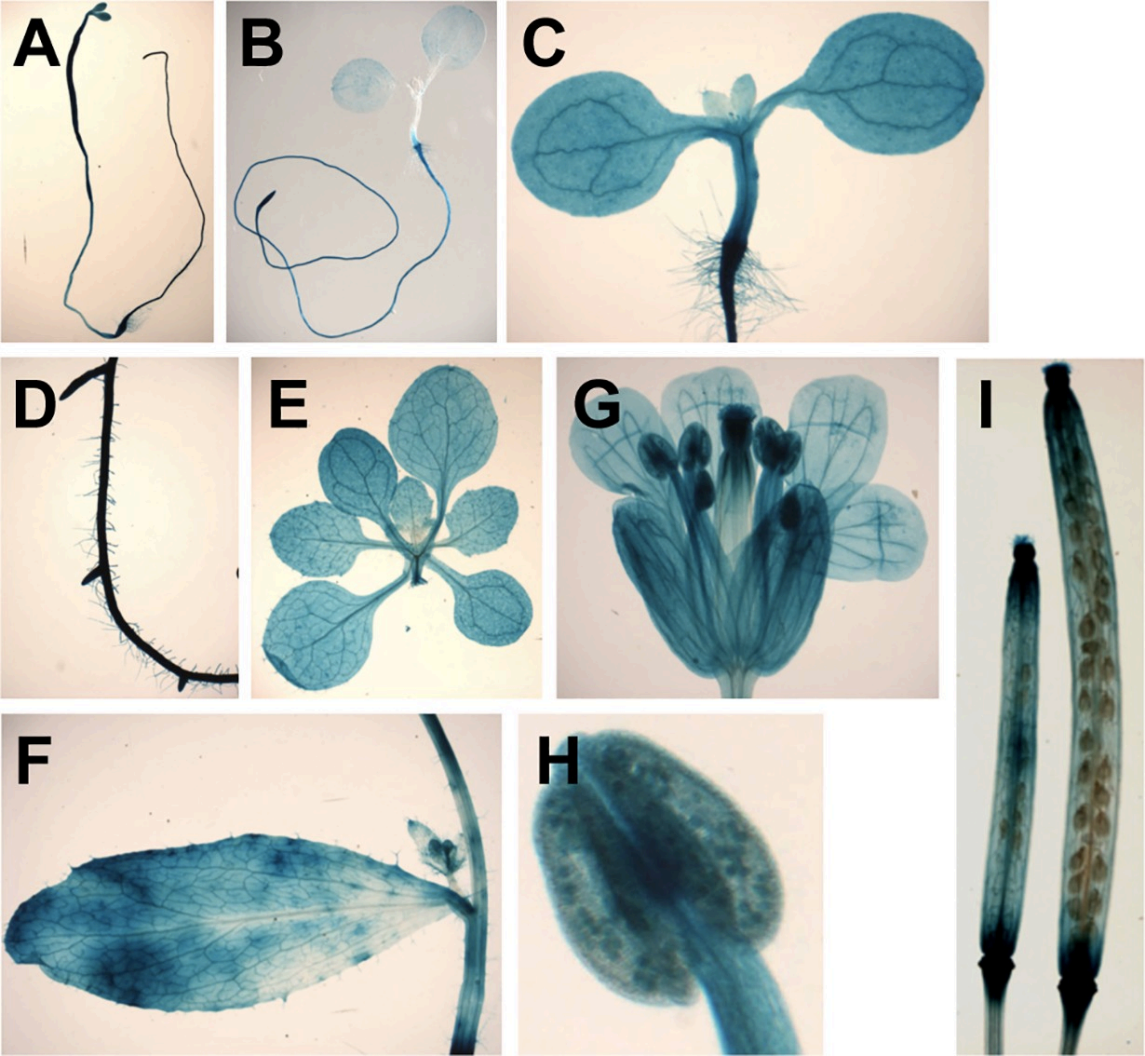
*BSK3* is broadly expressed. (A) A 6-day-old etiolated seedling. The apical hook of the seedling was manually opened to better observe cotyledon GUS staining. (B and C) Five-day-old light-grown seedlings. (D) Lateral roots of a 9-day-old seedling. (E) Rosette leaves of a 12-day-old plant. (F) Cauline leaf of a 24-day-old plant. (G) An open flower. (H) A close look at an anther shown in (G). (I) Siliques. GUS staining was performed at 37 ^o^C for 1 hour (B) or 24 hours (A and C-I).

To understand BSK3 function in BR-mediated plant growth and developmental processes, we examined *BSK3* gene expression using the *GUS* (β-glucuronidase) reporter gene driven by the native *BSK3* promoter. *BSK3* was expressed in the cotyledons, hypocotyls, and roots of etiolated and light-grown seedlings (Fig 6A–6C). Interestingly, roots exhibited higher *BSK3* expression than shoots in light-grown seedlings (Fig 6B). *BSK3* was also expressed in lateral roots (Fig 6D), rosette leaves (Fig 6E), and cauline leaves (Fig 6F). In flowers, *BSK3* was expressed in sepals, petals, stamens (including filaments, anthers, and pollens), and the top region of pistils (Fig 6G and 6H). In siliques, the top and basal regions exhibited higher *BSK3* expression (Fig 6I). Together, these results indicate that *BSK3* is broadly expressed in a variety of organs and tissues throughout plant development, consistent with the notion that *BSK3* may play an important role in BR-mediated plant growth and development.

### Increased dosage of BSK3 activates BR signaling, enhances plant growth, and confers defective shoot organ separation

**Fig 7:**
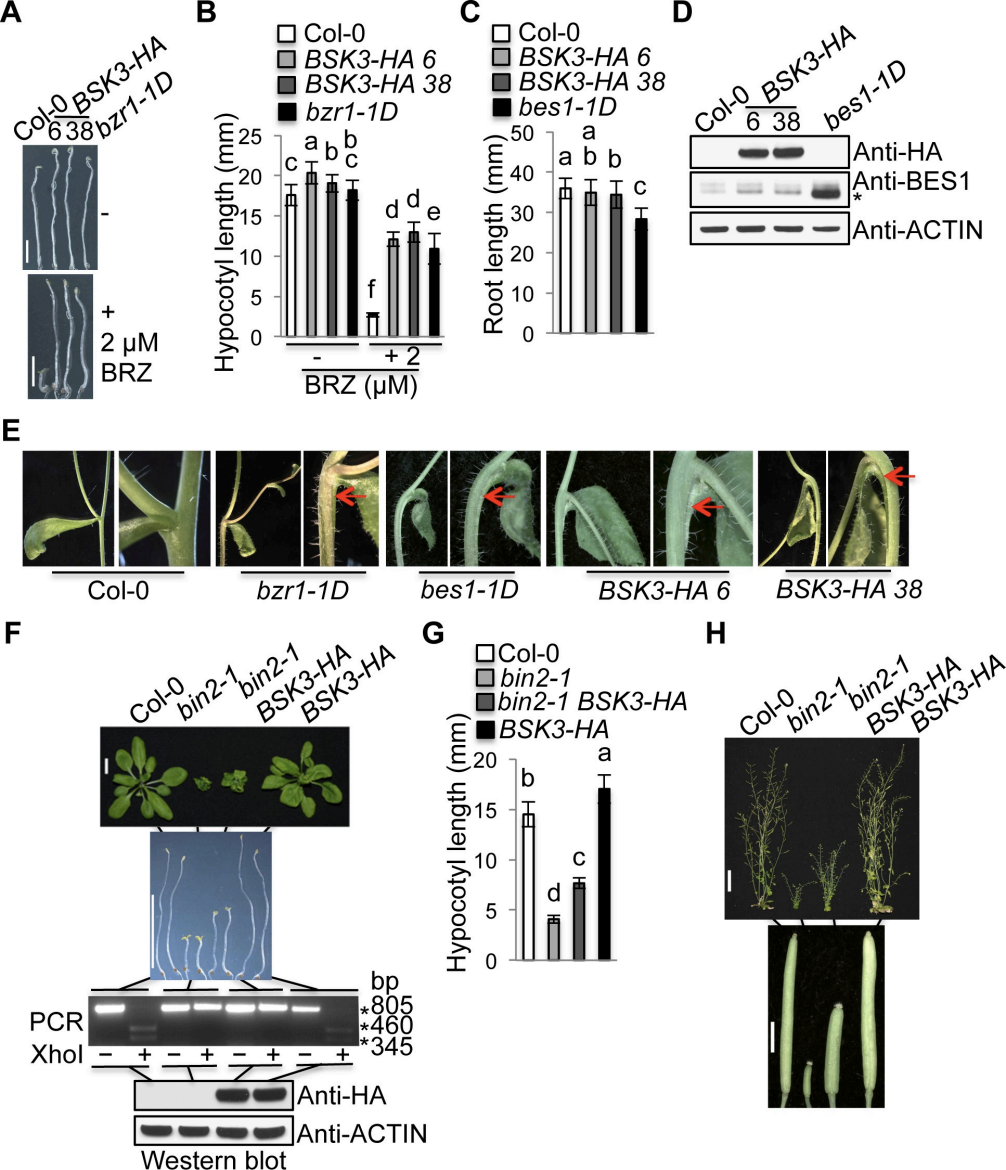
*BSK3-HA* plants exhibit increased BR responses and defective shoot organ separation. (A) Seven-day-old etiolated seedlings grown on ½ LS agar plates with or without 2 μM BRZ. Scale bar = 4 mm. (B) Hypocotyl length of 7-day-old etiolated seedlings. Error bars represent STD (n = 22–38). (C) Primary root length of 7-day-old light-grown seedlings. Error bars represent STD (n = 56–71). (D) Western blot analyses of BSK3-HA and BES1 protein expression. Asterisk (*) indicates the dephosphorylated BES1 protein. (E) Shoot organ fusion phenotypes of *bzr1-1D*, *bes1-1D*, and *BSK3-HA* plants. Arrows point to the fusion of cauline leaf and inflorescence stem. (F) Twenty-four-day-old plants and 7-day-old etiolated seedlings. *bin2-1* was genotyped by PCR using bin2-1-FW and bin2-1-RV primers. Col-0 PCR fragments have a XhoI site, while *bin2-1* PCR fragments do not have this site. Scale bar = 1 cm. (G) Hypocotyl length of 7-day-old etiolated seedlings. Error bars represent STD (n = 61–83). (H) Fifty-day-old plants and siliques. Scale bar = 4 cm (plants) or 2 mm (siliques). (D and F) Thirty micrograms of total proteins were loaded. (F-H) F3 or F4 *bin2-1* plants homozygous for the *BSK3pro*:*BSK3-HA* transgene were analyzed. (B, C, and G) Different letters above the bars indicate significant differences (P < 0.05).

To know the effects of *BSK3* gain-of-function on BR signaling and plant growth and development, we generated transgenic Arabidopsis plants that express an extra copy of *BSK3* gene under the control of the native *BSK3* promoter (*BSK3pro*:*BSK3-HA*). We screened forty-two independent transgenic lines, and lines 6 and 38 expressing high levels of BSK3-HA protein were selected for phenotypic analyses (Fig 7D). Etiolated *BSK3-HA* seedlings exhibited slightly increased hypocotyl growth and resistance to 2 μM BRZ (Fig 7A and 7B). Light-grown *BSK3-HA* seedlings did not exhibit obvious root growth phenotype, while *bes1-1D* seedlings exhibited reduced root growth (Fig 7C). *bes1-1D* is a new *bes1* mutant that we identified in the Col-0 background, which contains the same P233L mutation as the *bes1-D* mutant in the *Enkheim-2* (En-2) background [20]. *BSK3-HA* seedlings exhibited reduced BES1 protein phosphorylation, although considerably weaker than *bes1-1D* seedlings (Fig 7D), indicating increased BR signaling. Like *bzr1-1D* and *bes1-1D*, *BSK3-HA* plants exhibited cauline leaf and inflorescence stem fusion, causing the stem bending toward the axillary branch and cauline leaf (Fig 7E). Together, these results suggest that *BSK3-HA* plants exhibit increased BR responses and defective shoot organ separation.

To know whether increased *BSK3* expression could suppress the growth defects of *bri1-801* (GABI_227B07) and *bin2-1* mutants, we crossed the *BSK3pro*:*BSK3-HA* line 38 transgene into these mutants. Western blots using an anti-BRI1 antibody were not able to detect the full-length BRI1 protein in *bri1-801* (S7B Fig). If a truncated BRI1 protein is produced, this mutant protein would lack the region after LRR19, including the BR-binding island domain, transmembrane domain, and kinase domain [46]. Therefore, this mutant BRI1 protein cannot perceive BRs to initiate BR signaling. Like the *bri1-116* null mutant [4, 24], *bri1-801* plants exhibited severe growth defects (S7C–S7F Fig). All these results suggest that *bri1-801* is a null mutant. Consistent with the previous findings that *BSK3* overexpression from the *35S* promoter partially suppresses the dwarf phenotype of *bri1-116* [15], *BSK3-HA* expression from the native *BSK3* promoter also partially suppressed the dwarf phenotype of *bri1-801* (S7C and S7F Fig). In addition, our results further revealed that *BSK3-HA* expression partially suppressed all examined aspects of the growth defects of *bri1-801*, including root growth, shoot growth, and male fertility (S7C–S7F Fig). These results indicate that BSK3 can activate BR signaling without a functional BRI1 receptor, and it is an important player that regulates BRI1-mediated plant growth and developmental processes.

The homozygous *bin2-1* mutant exhibits severe *bri1*-like growth defects, such as dwarf and male sterility [34]. A previous study reported that *BSK3* overexpression from the *35S* promoter could not suppress the dwarf phenotype of *bin2-1* [15]. However, *BSK3-HA* expression from the native *BSK3* promoter partially suppressed the growth defects of *bin2-1*. Etiolated *bin2-1 BSK3-HA* seedlings exhibited longer hypocotyls than those of *bin2-1* seedlings (Fig 7F and 7G), and mature *bin2-1 BSK3-HA* plants were bigger than *bin2-1* plants (Fig 7F and 7H). Although smaller than those of wild-type, the siliques of *bin2-1 BSK3-HA* plants were larger than those of *bin2-1* plants and could set seeds (Fig 7H). Our findings demonstrate that *BSK3-HA* expression can partially rescue the growth defects of the *bin2-1* mutant, including shoot growth and male fertility.

### *BSK3-HA* expression upregulates *BSU1* transcript and protein levels

**Fig 8:**
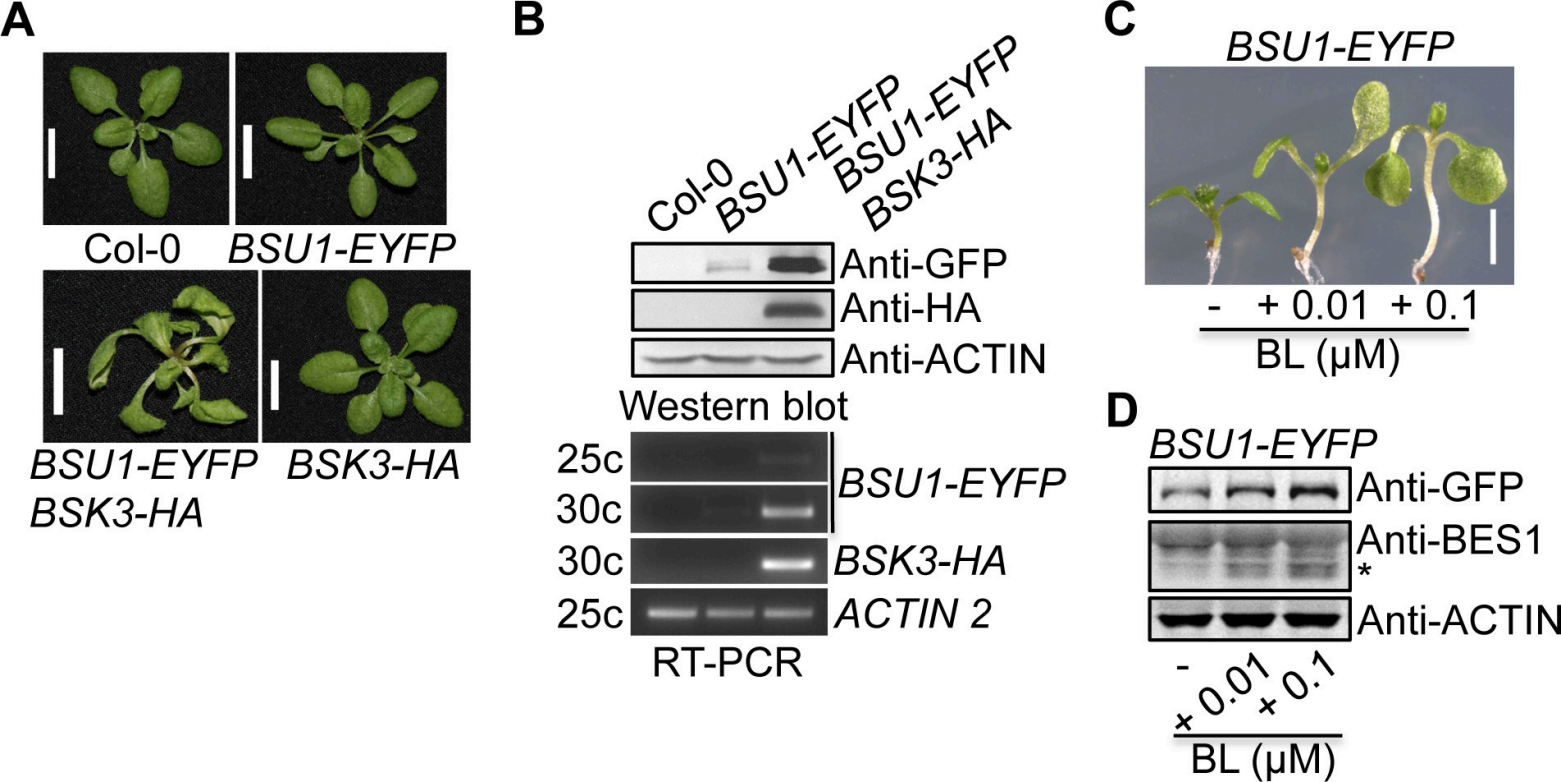
*BSU1-EYFP BSK3-HA* plants exhibit increased *BSU1* transcript and protein levels. **(**A) Twenty-two-day-old plants. Transgenic plants hemizygous for the *35Spro*:*BSU1-eYFP* and *BSK3pro*:*BSK3-HA* transgenes were analyzed. Scale bar = 1 cm. (B) Semi-quantitative RT-PCR and western blot analyses of *BSU1-EYFP* and *BSK3-HA* gene and protein expression. One hundred micrograms of total proteins from 7-day-old light-grown seedlings were loaded. Transgenic plants homozygous for the *BSK3pro*:*BSK3-HA* transgene and hemizygous for the *35Spro*:*BSU1-EYFP* transgene were analyzed. (C) Seven-day-old light-grown seedlings grown on ½ LS agar plates with or without BL. Scale bar = 2.5 mm. (D) Western blot analyses of BSU1-EYFP and BES1 protein expression in seedlings shown in (C). Fifty micrograms of total proteins were loaded. Asterisk (*) indicates the dephosphorylated BES1 protein.

When we tried to test BSK3 and BSU1 protein interaction in Arabidopsis by co-IP, we crossed the *BSK3pro*:*BSK3-HA* line 38 transgene into *35Spro*:*BSU1-EYFP* plants. *BSU1-EYFP* overexpression plants did not exhibit obvious growth phenotypes, looking like the wild-type plants (Fig 8A). However, we noticed that F1 plants co-expressing *BSK3-HA* and *BSU1-EYFP* exhibited *bes1-1D*-like long and twisted rosette leaves (Fig 8A) [20], indicating dramatically increased BR responses. To examine BSK3-HA and BSU1-EYFP protein expression, we performed western blots using anti-HA and anti-GFP antibodies, respectively. Interestingly, BSU1-EYFP protein levels were significantly increased in *BSK3-HA BSU1-EYFP* plants relative to those of *BSU1-EYFP* plants (Fig 8B). To further determine whether increased BSU1-EYFP protein levels are caused by increased *BSU1-EYFP* transcripts, we performed semi-quantitative RT-PCR using primers specific for *BSU1-EYFP* transcripts. *BSU1-EYFP* transcript levels were significantly increased in *BSK3-HA BSU1-EYFP* plants than those of *BSU1-EYFP* plants (Fig 8B). Since *BSU1-EYFP* transcription is under the control of the *35S* promoter, increased *BSU1-EYFP* transcript levels in *BSK3-HA BSU1-EYFP* plants suggest that BSK3 is involved in post-transcriptional regulation of *BSU1-EYFP* transcript levels.

Our findings revealed that BSK3 is involved in upregulating BSU1 protein levels to activate BR signaling. To determine whether BRs can upregulate BSU1 protein levels, we treated light-grown *BSU1-EYFP* seedlings with 0.01 and 0.1 μM BL and examined BSU1-EYFP protein levels by western blots. BL-treated *BSU1-EYFP* seedlings exhibited increased hypocotyl growth (Fig 8C), indicating increased BR responses. Consistent with increased BR responses, BL-treated *BSU1-EYFP* seedlings exhibited reduced BES1 phosphorylation and increased BSU1-EYFP protein levels (Fig 8D). These results demonstrate that BRs upregulate BSU1 protein levels to activate BR signaling.

## Discussion

Since their discovery in 2008 [15], the functions of the BSK family proteins in the BR signaling pathway have remained elusive. In this study, we focus on BSK3 and elucidate its function in BR signaling and plant growth and development. Our genetic screen identified a G2R missense mutation in the *bsk3-2* mutant, causing *BSK3* loss of function and decreased BR responses (Fig 1A–1D). Interestingly, the G2R mutation blocked BSK3 myristoylation and plasma membrane localization (Fig 2B–2D). These results suggest that plasma membrane localization is required for BSK3 function in BR signaling. In addition to the G2R mutation, our genetic screen also identified three additional missense mutations (R156K/*bsk3-3*, G226E/*bsk3-4*, and G238S/*bsk3-6*) in BSK3 kinase domain, causing *BSK3* loss-of-function and decreased BR responses (Figs 1D and 3A). The arginine residue corresponding to BSK3 R156 is absolutely conserved in all BSK family members, while the glycine residue corresponding to BSK3 G226 is also highly conserved in all BSK family members except BSK11 (S2 Fig). Expression of BSK3^R156K^-HA and BSK3^G226E^-HA proteins under the control of the native *BSK3* promoter in the *bsk3-1* mutant conferred defective shoot growth, including smaller rosette leaves (Fig 3E), reduced plant height (Fig 3G), and smaller siliques (Fig 3H). These results suggest that BSK3^R156K^-HA and BSK3^G226E^-HA proteins may have dominant-negative effects, which interfere with the function of additional BSK family members. These mutant proteins may be non-functional and sequester BSK interactors, causing BSK loss-of-function. Unlike R156K and G226E, the G238S mutation appeared to destabilize BSK3 protein to reduce its levels in the *bsk3-6* mutant, causing BSK3 loss-of-function (Fig 3I). The glycine residue corresponding to BSK3 G238 is absolutely conserved in all BSK family members (S2 Fig). It will be interesting to know whether this glycine regulates the protein stability of other BSK family members. Together, our results demonstrate that the kinase domain is crucial for BSK3 function in BR signaling.

The TPR domain is a 34 amino acid structural motif involved in mediating protein-protein interaction and assembling a multiprotein complex, which has been found in a wide variety of proteins in all organisms [47–49]. Previous studies have shown that TPR motifs are crucial for BSK1 and SSP/BSK12 functions in regulating plant defense responses and suspensor development during embryogenesis, respectively [29, 41]. A recessive R443Q missense mutation in the TPR motifs confers BSK1 loss-of-function and defective defense responses [41]. TPR deletion completely inactivates SSP/BSK12, causing defective suspensor development during embryogenesis [29]. More recently, TPR motifs were reported to negatively regulate OsBSK3 activity in BR signaling, as *OsBSK3*^*TPR-Δ*^*-GFP* overexpression from the *35S* promoter activates BR signaling more efficiently than *OsBSK3-GFP* overexpression [44]. We demonstrate that the C-terminal three tandem TPR motifs are not essential for BSK3 function in BR signaling. However, TPR deletion impairs BSK3 function, suggesting that TPR motifs are required for the full function of BSK3 in BR signaling (Fig 5C). Therefore, different from the negative role of TPR motifs in the control of OsBSK3 activity in BR signaling [44], our results do not support a negative role of TPR motifs in regulating BSK3 activity in BR signaling. Interestingly, TPR deletion impairs BSK3/BSK3 interaction and BSK3/BSK1 interaction (Fig 5D), suggesting that TPR motifs contribute to BSK homodimer/heterodimer formation.

GSK3 kinases are highly conserved in eukaryotes and regulate diverse physiological and developmental processes [50, 51]. In the BR signaling pathway, the GSK3-like kinase BIN2 phosphorylates BZR1 and BES1 transcription factors to inhibit BR signaling via multiple mechanisms, causing proteasomal degradation of BZR1 and BES1, inhibiting their DNA binding, and promoting their binding to 14-3-3 proteins to cause cytoplasmic retention of BZR1 and BES1 [19, 21, 52, 53]. We found that BSK3 directly interacted with BIN2 in Arabidopsis, and their interaction did not appear to be regulated by BRs (Fig 4C and 4D). Furthermore, BSK3 contained nine putative GSK3 consensus phosphorylation motifs and was a BIN2 kinase substrate (Fig 4F and 4G) [30]. Interestingly, BIN2 phosphorylation of BSK3 enhanced BSK3 interactions with BSK1, BSK3, BRI1, and BSU1 (Fig 4H). These results suggest that BIN2 may have a positive role in BR signaling, promoting BSK homodimer/heterodimer formation, BSK3/BRI1 interaction, and BSK3/BSU1 interaction to enhance BR signaling. A similar positive role of GSK3 kinase in Wnt signaling was reported. GSK3 kinase phosphorylates and activates the Wnt co-receptor low-density lipoprotein receptor-related protein 6 (LRP6) to activate Wnt signaling [54].

The BSU1 protein family is composed of four members (BSU1, BSU1-LIKE 1/BSL1, BSL2, and BSL3) and positively regulate BR signaling [14, 16, 55]. BSU1 phosphatase inactivates BIN2 kinase activity by dephosphorylating the phosphotyrosine residue pTyr200 of BIN2 to activate BR signaling [16]. We and others have shown that BSK3 and BSK1 physically interact with BSU1 and maybe other BSU1 family members (BSL1, BSL2, and BSL3) to regulate BR signaling [16, 55]. However, the molecular mechanisms underlying BSK-mediated BSU1 activation is still not known. We found that *BSK3-HA* expression driven by the native *BSK3* promoter upregulated *BSU1-EYFP* transcript levels via a post-transcriptional mechanism to increase BSU1 protein levels and thereby activate BR signaling (Fig 8B). These results reveal that an important mechanism to activate BR signaling is BSK3-mediated upregulation of *BSU1* transcript and protein levels. The detailed mechanisms require further investigation.

Our genetic analyses showed that all five *bsk3* loss-of-function mutants that we identified are caused by semidominant mutations (Fig 1 and S1 Fig). Interestingly, the *bsk3-1* T-DNA loss-of-function mutant is also semidominant. Homozygous *bsk3-1* seedlings exhibited stronger BR resistance than heterozygous *bsk3-1* seedlings (Fig 1H and 1I), suggesting a dose-dependent effect of BSK3 protein on BR responses. Expression of a kinase dead BSK3^K86R^-HA protein under the control of the native *BSK3* promoter fully rescued the root growth defect and partially rescued the BR resistance phenotype of the *bsk3-1* mutant, suggesting that this mutant protein can still activate BR signaling to promote root growth (Fig 3D). These results provide experimental evidence to support that BSK3 may function as a scaffold protein to positively regulate BR signaling. Scaffold proteins are signal-organizing proteins that regulate a variety of signal transduction pathways. Their basic functions include assembling their interactors into specific protein complexes and localizing their interactors to specific subcellular compartments [56, 57]. As a scaffold protein in the BR signaling pathway, BSK3 may function as monomers, homodimers, and/or heterodimers with other BSK family members. BSK3 may sequester BSU1 phosphatase and BIN2 kinase at the inner face of the plasma membrane to enhance their interaction [16]. BSK3 may also enhance the oligomerization of the BSU1 family proteins [58]. In either case, BSU1 phosphatase may dephosphorylate the phosphotyrosine residue pTyr200 of BIN2 more efficiently to inactivate BIN2 kinase and thereby activate BR signaling.

Despite extensive efforts, we were not able to detect BSK3 autophosphorylation in kinase assay reactions containing either Mg^2+^ or Mn^2+^ (Fig 3B), indicating that unlike BRI1 receptor kinase, BSK3 does not have kinase activity under our conditions. However, these results could not rule out that BSK3 may have kinase activity *in planta*. Supporting this hypothesis, the kinase dead K86R mutation impairs BSK3 function in BR signaling. Expressed at similar levels, BSK3^K86R^-HA protein was less efficient to rescue the BR resistance phenotype of *bsk3-1* seedlings than BSK3-HA protein (Fig 3C and 3D), suggesting that BSK3 kinase activity may be required for its full function in BR signaling. The mechanisms underlying BSK3 kinase activation *in planta* are not known. Based on the structural studies of BSK8 kinase domain [40], one possibility is that BSK3 binding to other proteins may reverse the catalytically inactive auto-inhibition state of BSK3 to a catalytically active state. Alternatively, BSK3 phosphorylation by BRI1 receptor kinase may activate BSK3 kinase activity. This hypothesis is supported by the finding that OsBSK3 phosphorylation by OsBRI1 receptor kinase appears to enhance OsBSK3 autophosphorylation *in vitro* [44]. Together, our findings suggest that BSK3 may function as a scaffold protein with possible kinase activity to regulate BR signaling. Future research to demonstrate BSK3 kinase activity, investigate BSK3 kinase activation mechanisms, and identify BSK3 physiological substrates in the BR signaling pathway will advance the understanding of BR signaling mechanisms. Alternatively, our results could not rule out that BSK3 may not function as a kinase. However, full BSK3 function in BR signaling requires ATP binding, which may modulate the scaffold function of BSK3. Supporting this hypothesis, some pseudokinases have been shown to bind ATP, which may have a structural or functional role in regulating pseudokinase activity in the absence of catalytic activity [59].

The BSK protein family is composed of twelve members. Previous studies using a loss-of-function approach to understand their role in BR-mediated plant growth and development were not successful [15, 30]. Despite reduced growth, *bsk3/4/6/7/8* pentuple mutants do not exhibit dark green and rounded leaves, male sterility, and reduced silique growth, which are typical growth phenotypes of BR deficient and response mutants [3, 30–33]. We performed phenotypic analyses on 10 *bsk* T-DNA insertion mutants (*bsk1*, *2*, *3*, *4*, *5*, *6*, *8*, *10*, *11*, and *12*), including growth phenotypes and responses to 0.01 and 0.1 μM BL (S1 Table). None of these mutants exhibited obvious growth phenotypes and BR resistance except that the *bsk3-1* mutant exhibited slightly reduced root growth and BR resistance (Fig 1I and S6 Fig). Supporting an important function of BSK3 in root growth, *BSK3* was highly expressed in seedling roots (Fig 6B). In addition, all five *bsk3* mutants that we identified exhibited slightly reduced root growth (S6A and S6B Fig). Our BSK3 gain-of-function approach revealed that in addition to root growth, BSK3 is also important for various shoot growth and developmental processes. *BSK3-HA* expression driven by the native *BSK3* promoter conferred increased seedling hypocotyl growth and cauline leaf and inflorescence stem fusion (Fig 7A, 7B and 7E). In addition, *BSK3-HA* expression partially suppressed the growth defects of *bri1-801* and *bin2-1* mutants, including root growth, shoot growth, and male fertility (Fig 7F–7H and S7C–S7F Fig). Interestingly, *BSK3*^*R156K*^*-HA* or *BSK3*^*G226E*^*-HA* expression under the control of the native *BSK3* promoter in the *bsk3-1* mutant conferred reduced rosette leaf growth, plant height, and silique growth (Fig 3E–3H). Together, our findings demonstrate that BSK3 plays an important role in BR-mediated plant growth and development, including root growth, shoot growth, and organ separation. Future research to generate mutant plants loss of all twelve *BSK* genes may provide further evidence in support of the crucial role of the BSK family proteins in BR-mediated plant growth and development.

## Materials and methods

### Plant materials and growth conditions

All *Arabidopsis thaliana* mutants and transgenic lines were in the Columbia (Col) ecotype. Plants were grown under long-day conditions (16 h light/8 h dark) at 22 ^o^C. Seeds were surface sterilized in a solution containing 30% bleach and 0.04% triton X-100 for 15 minutes, washed 3 times in sterile water, and cold treated for 3 days at 4 ^o^C to synchronize germination. Seedlings were grown on ½ LS (Linsmaier and Skoog medium, Caisson Labs) plates containing 1% sucrose and 0.8% agar.

### Transgenic plant lines

To make a *BSK3pro*:*GUS* construct, *BSK3* promoter was cloned into pENTR/D-TOPO using the pENTR directional TOPO cloning kit (Invitrogen). *BSK3* promoter was recombined into pGWB203 (GUS) [60] using the Gateway LR clonase II enzyme mix (Invitrogen). To make *BSK3pro*:*BSK3*^*WT/K86R/R156K/G226E/G238S*^*-HA*, *BSK3pro*:*BSK3-GFP*, and *BES1pro*:*BES1-HA* constructs, *BSK3* or *BES1* genomic DNA containing the promoter and coding sequence without the stop codon was cloned into pENTR/D-TOPO. Site-directed mutagenesis PCRs were performed to create BSK3^K86R/R156K/G226E/G238S^ mutations. To make a *BSK3pro*:*BSK3*^*TPR-Δ*^*-HA* construct, *BSK3* genomic DNA containing the promoter and partial coding sequence that does not encode three TPR motifs was cloned into pENTR/D-TOPO. All above inserts were recombined into pEarleyGate 301(HA) [61] or pGWB204 (GFP) [60]. To make *BSK3pro*:*BSK3*^*WT/G2A/G2R*^*-mCitrine* constructs, *BSK3* promoter, full-length cDNA without the stop codon containing the *WT*/*G2A*/*G2R* mutations, and the *mCitrine* coding sequence were cloned into pDONR-P4P1R, pDONR221, and pDONR-B2RB3, respectively. These three DNA fragments were assembled into pB7m34GW [62] using the Gateway LR clonase II Plus enzyme mix (Invitrogen). To make *BSK1*, *CDG1*, *BSU1*, and *BIN2* overexpression constructs, the full-length cDNA without the stop codon of each gene was cloned into pENTR/D-TOPO. All inserts were recombined into pK7YWG2 (EYFP*)* [62]. All binary vectors were introduced into *Agrobacterium tumefaciens* strain GV3101 (helper plasmid pMP90) by electroporation. The floral dip method was used to transform Arabidopsis plants [63]. Transgenic plants were selected on ½ LS agar plates containing 0.01% Basta (Finale herbicide, Bayer CropScience), hygromycin (25 μg/ml), or 50 μg/ml kanamycin without sucrose.

### Genetic screen and positional cloning

EMS-mutagenized M2 seeds (Catalog nos. M2E-02-05 and M2E-01A-07, ~ 448100 M2 seeds from ~ 56012 M1 plants) and activation-tagged lines (Stock no. CS31100, ~ 62000 lines) were purchased from Lehle Seeds and Arabidopsis Biological Resource Center, respectively. Sterilized seeds were grown on ½ LS agar plates containing 0.1 μM brassinolide (BL) (Daiichi Fine Chemical). Seven-day-old light-grown BL resistant seedlings exhibiting long roots were selected. All candidate mutants were retested for BL resistance in the next generation. The semi-dominant *brr1* mutant was crossed into the *Landsberg erecta* (Ler) ecotype to create a mapping population. DNA extractions were performed using a CTAB method [64] from BL sensitive F2 plants, and SSLP (simple sequence length polymorphism), INDEL (insertion-deletion polymorphism), and CAPS (cleaved amplified polymorphic sequence) markers were used to map the *BSK3* mutation in the *brr1* mutant by PCRs.

### Co-immunoprecipitation (Co-IP) and western/far-western blots

Total protein extracts were prepared from Arabidopsis seedlings using an extraction buffer containing 50 mM Tris·HCl (pH 7.5), 150 mM NaCl, and 0.5% Igepal CA-630. Microsomal proteins were prepared from Arabidopsis seedlings or *Nicotiana benthamiana* leaves as described previously [65]. The complete EDTA-free protease inhibitor cocktail (Roche Applied Science) was added to all buffers. All centrifugation steps were carried out at 4 ^o^C. For protein fractionation to isolate microsomal proteins, total protein extracts were centrifuged for 30 minutes at 5000 × g, and supernatants were then centrifuged for 1 hour at 100000 × g. Co-IP and western blots were performed as previously described with slight modification [66]. Protein G agarose beads (Roche Applied Science) were used to collect the immune complexes. The tris-buffered saline buffer (TBST, 0.05% tween 20, pH7.6) was used for western blots. Proteins were detected using the SuperSignal West Pico or Dura chemiluminescent substrates (Thermo Scientific).

Far-western blot assays were performed to examine BSK3 interactions with BSK3, BSK1, BRI1, and BSU1. MBP-BSK3, GST-BIN2, and GST-BIN2^K69R^ fusion proteins were expressed in *E*. *coli* BL21 (DE3) cells and purified using amylose resin (New England Biolabs) and glutathione sepharose 4B beads (GE Healthcare Life Sciences), respectively, according to the manufacturer's instructions. MBP-BSK3 was incubated with GST-BIN2 or GST-BIN2^K69R^ in 100 μl kinase buffer (20 mM Tris-HCl [pH 7.5], 10 mM MgCl_2_, 5 mM dithiothreitol, and 100 μM ATP) for 2 hours at 30 ^o^C. After kinase assays, proteins were analyzed by SDS-PAGE and transferred onto nitrocellulose membranes. Nitrocellulose membranes were stained with ponceau S to detect MBP-BSK3 and GST-BIN2 or GST-BIN2^K69R^ proteins. Nitrocellulose membrane strips containing only MBP-BSK3 without GST-BIN2 or GST-BIN2^K69R^ were incubated with 100 μl of GST fusion proteins overnight at 4 ^o^C. GST-BSK3, GST-BSK1, GST-BRI1-KD, and GST-BSU1 fusion proteins were synthesized using the TNT SP6 high-yield wheat germ protein expression system (Promega). GST fusion proteins bound to MBP-BSK3 were detected by an HRP-conjugated anti-GST antibody (GE Healthcare Life Sciences).

### RT-PCR

RNAs were prepared using the Spectrum™ plant total RNA kit (Sigma). An on-column DNase I digest set (Sigma) was used to remove contaminating DNA. Complementary DNAs (cDNAs) were synthesized using the SuperScript III first-strand synthesis system (Invitrogen). RT-PCR reactions were carried out using gene specific primers, and PCR products were analyzed by agarose gel electrophoresis.

### *In vitro* myristoylation assays

*BSK3* full-length cDNA without the stop codon was cloned into pTNT (HA) to generate a C-terminal HA epitope-tagged BSK3 construct. Site-directed mutagenesis PCRs were performed to create BSK3^G2A/G2R^ mutations. Proteins were synthesized using the TNT SP6 high-yield wheat germ protein expression system. [^3^H]Myristic acid (tetradecanoic acid) (PerkinElmer) was added to the reactions at a concentration of 0.5 μCi/μl for radiolabelling. Reaction products were analyzed by SDS-PAGE, western blot, and fluorography.

### *In vitro* kinase assays

*BIN2* or *BSK3* full-length cDNA was cloned into pENTR/D-TOPO. Similarly, *BRI1* partial cDNA encoding the kinase domain containing a stop codon (*BRI1-KD*) was cloned into in the same vector. Site-directed mutagenesis PCRs were performed to create BSK3^K86R^, BIN2^K69R^, and BRI1-KD^K911E^ kinase dead mutations. All DNA fragments were recombined into pTNT (GST) or pDEST15 (GST) to generate N-terminal GST-tagged fusion protein constructs. GST fusion proteins were synthesized using the TNT SP6 high-yield wheat germ protein expression system or expressed in *E*. *coli* BL21 (DE3) cells induced by 1 mM IPTG for 4 hours at 30 ^o^C. All GST fusion proteins were purified using glutathione sepharose 4B beads according to the manufacturer's instructions. Purified GST fusion proteins were incubated in 50 μl kinase buffer (20 mM Tris-HCl [pH 7.5], 10 mM MgCl_2_ or MnCl_2_, 5 mM dithiothreitol, and 100 μM ATP) containing 10 μCi [γ-^32^P]ATP (PerkinElmer) for 1 hour at 30 ^o^C or 2 hours at room temperature. Kinase reactions were terminated by adding 20 μL 4x SDS-PAGE sample buffer and boiled for 5 minutes. Reaction products were analyzed by SDS-PAGE and fluorography.

### GST pull-down assays

*BSU1* full-length cDNA was cloned into pENTR/D-TOPO and recombined into pTNT (GST) to generate an N-terminal GST-tagged fusion protein construct. *BSK1* full-length cDNA without the stop codon was cloned into pENTR/D-TOPO and recombined into pTNT (FLAG) to generate a C-terminal FLAG-tagged fusion protein construct. GST fusion proteins were synthesized using the TNT SP6 high-yield wheat germ protein expression system and purified using glutathione sepharose 4B beads. GST pull-down assays were performed in the PBS buffer (pH 7.4) containing 1 mM dithiothreitol and 0.1 to 1% igepal CA-630 at 4 ^o^C. After 2 hours of incubation of GST fusion proteins and HA- or FLAG-tagged proteins, beads were washed 4 times with the same buffer. Finally, Beads were resuspended in 1x SDS-PAGE sample buffer and boiled for 5 minutes. The supernatants were analyzed by SDS-PAGE and western blots using anti-GST, anti-HA, and anti-FLAG antibodies.

### Bimolecular fluorescence complementation (BiFC) assays

The full-length or partial cDNA of *BSK3*, *BSK3*^*TPR-Δ*^, *BSK1*, *BSK1*^*TPR-Δ*^, *BRI1*, *BSU1*, *BIN2*, or *TMK1* without the stop codons in pENTR/D-TOPO was recombined into pSPYNE-35S and pSPYCE-35S [67] to generate BiFC expression constructs. These constructs were introduced into *Agrobacterium tumefaciens* strain GV3101 (helper plasmid pMP90) by electroporation. BiFC assays were performed in an *Nicotiana benthamiana* transient expression system as previously described [68]. Confocal microscopy was performed with the Leica DM IRE2 inverted microscope.

### GUS assays

GUS staining of plant tissues was performed at 37 ^o^C in a solution containing 100 mM sodium phosphate (pH 7.0), 10 mM EDTA, 0.5 mM K_4_Fe[CN]_6_, 0.5 mM K_3_Fe[CN]_6_, 0.1% triton X-100, and 1 mM X-Gluc (5-Bromo-4-chloro-3-indoxyl-beta-D-glucuronide cyclohexylammonium salt) (Gold Biotechnology). GUS-stained tissues were incubated in 70% ethanol to remove chlorophyll and cleared in the Hoyer's solution. GUS expression patterns were imaged with a Leica MZ FLIII fluorescent dissecting microscope using the imaging software Leica Application Suite V4.0.

### Statistical analyses

All statistical analyses were carried out by analysis of variance (ANOVA) with the JMP Pro 13.1 software (SAS Institute). Tukey’s HSD (honestly significant difference) test results were grouped by letters, indicating measurements that were not significantly different at 95% confidence. Different letters indicate significant differences (P < 0.05).

### Accession numbers

Arabidopsis Genome Initiative locus identifiers for the genes employed in this study are as follows: *BSK1* (At4g35230), *BSK2* (At5g46570), *BSK3* (At4g00710), *BSK4* (At1g01740), *BSK5* (At5g59010), *BSK6* (At3g54030), *BSK7* (At1g63500), *BSK8* (At5g41260), *BSK9* (At3g09240), *BSK10* (At5g01060), *BSK11* (At1g50990), *BSK12/SSP* (At2g17090), *BRI1* (At4g39400), *BSU1* (At1g03445), *CDG1* (At3g26940), *BES1* (At1g19350), *BIN2* (At4g18710), *TMK1* (At1g66150), and *ACTIN 2* (At3g18780).

## Supporting information

S1 Fig Isolation of brassinosteroid resistant mutants. (A-E) Root growth of Col-0 and *brr* mutants (+/- BL). Seedlings were grown in the light for 7 days. Error bars represent SEM (n = 42–64).
(TIF)

S2 Fig BSK3 mutations in the kinase domain. The kinase domains of the BSK family proteins were predicted by SMART. Protein sequence alignment was performed using Clustal Omega (http://www.ebi.ac.uk/Tools/msa/clustalo/).
(PDF)

S3 Fig BSK3 interacts with BSK3, BSK1, BRI1, BSU1, and BIN2. GST pull-down assays detecting BSK3-HA interactions with GST-BSK3 (A), GST-BRI1-KD (C), GST-BSU1 (D), and GST-BIN2 (E) and BSK1-FLAG and GST-BSK3 interaction (B). GST, GST fusion proteins, BSK3-HA, and BSK1-FLAG synthesized by the TNT SP6 high-yield wheat germ protein expression system were used for GST pull-down assays.
(TIF)

S4 Fig Western blot analyses of transiently expressed proteins in *Nicotiana benthamiana* leaves. (A and B) Twenty-five micrograms of microsomal proteins were loaded. Unknown proteins stained with Ponceau S are shown as loading controls.
(TIF)

S5 Fig TPR deletion BSK3 interacts with BRI1, BIN2, and BSU1. (A). BiFC assays in *Nicotiana benthamiana* leaves. Scale bar = 50 μm. (B and C) Western blot analyses of transiently expressed proteins in *Nicotiana benthamiana* leaves. Twenty-five micrograms of microsomal proteins were loaded. Unknown proteins stained with Ponceau S are shown as loading controls.
(TIF)

S6 Fig The *bsk3* mutants exhibit slight root growth defects and do not exhibit obvious shoot growth defects. (A and B) Primary root length of 7-day-old light-grown seedlings. (C and D) Hypocotyl length of 7-day-old etiolated and light-grown seedlings. (E) Rosette leaves of 4-week-old plants. Primary bolts were removed to better observe rosette leaves. (F) Forty-eight-day-old plants. (A-D) Different letters above the bars indicate significant differences (P < 0.05). Error bars represent STD (n = 47–91).
(TIF)

S7 Fig Increased *BSK3* expression partially suppresses the growth defects of the *bri1-801* mutant. (A) The gene structure of *BRI1*. White boxes represent 5’ UTR and 3’ UTR, and gray box represents exon. Triangle represents the T-DNA insert, and the insertion site is between 1668 bp and 1669 bp in the exon of *BRI1*. (B) Western blot analysis of BRI1 protein expression. Twenty micrograms of microsomal proteins from 9-day-old light-grown seedlings were loaded. BRI1 was detected by an anti-BRI1 antibody. An unknown protein recognized by the anti-BRI1 antibody is shown as a loading control. (C) Twenty-two-day-old plants and etiolated 7-day-old seedlings. PCR primers used for genotyping *bri1-801* are shown in (A). Thirty micrograms of total proteins were loaded for western blots. BSK3-HA and ACTIN proteins were detected by anti-HA and anti-ACTIN antibodies, respectively. (D) Hypocotyl length of 7-day-old etiolated seedlings. (E) Primary root length of 7-day-old light-grown seedlings. (F) Fifty-day-old plants and siliques. (D and E) Different letters above the bars indicate significant differences (P < 0.05). Error bars represent SD (n = 41–75).
(TIF)

S1 Table T-DNA insertion mutants of the *BSK* family genes. (PDF)

S2 Table PCR primers used in this study. (PDF)
