# Supplementary figures and images for: BRASSINOSTEROID-SIGNALING KINASE 3, a plasma membrane-associated scaffold protein involved in early brassinosteroid signaling
Source: PLoS Genet. 2019 Jan 7;15(1):e1007904. doi: 10.1371/journal.pgen.1007904 (PMC6336344; doi:10.1371/journal.pgen.1007904)

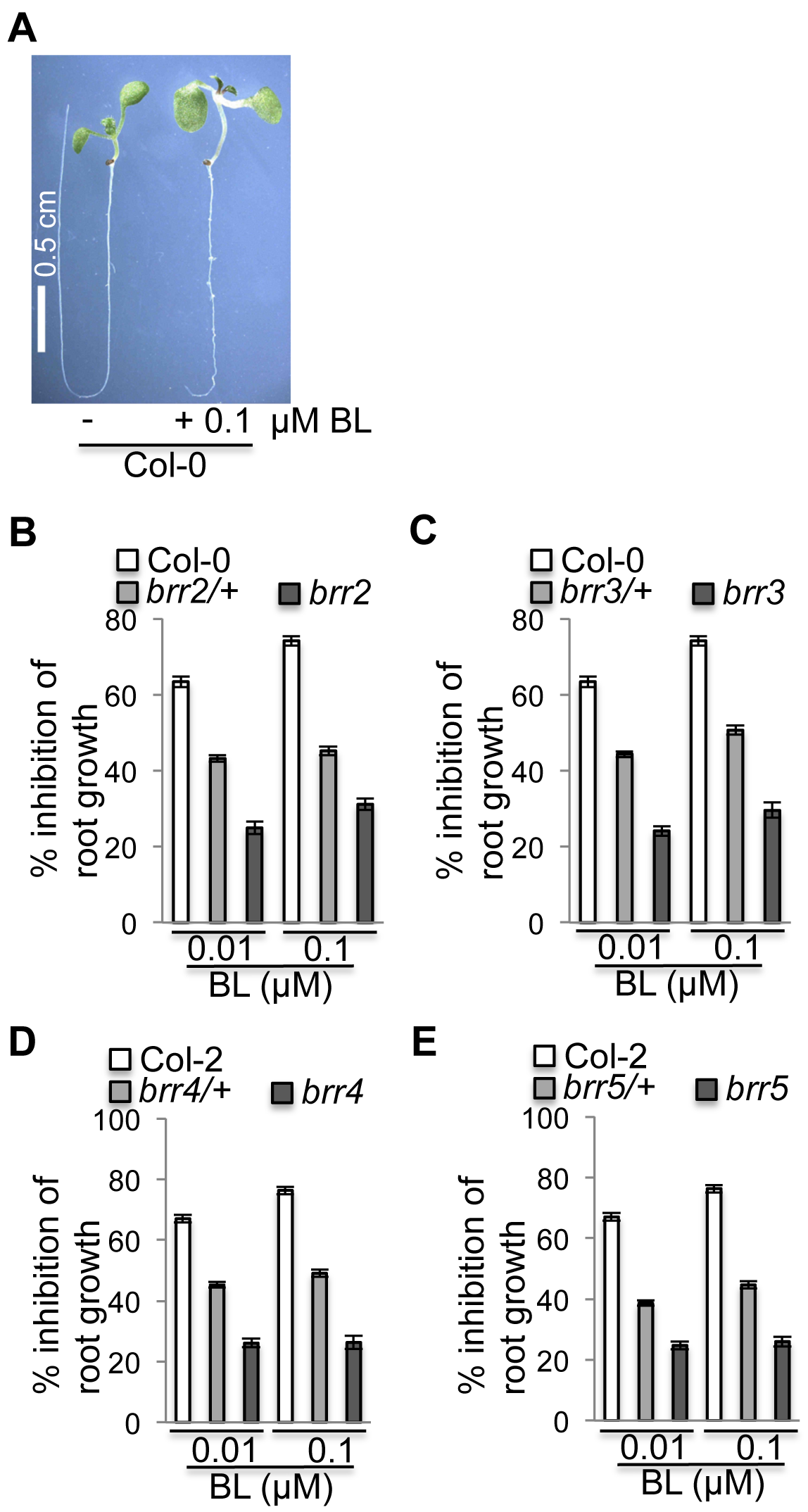

Supplement: S1 Fig — (A-E) Root growth of Col-0 and brr mutants (+/- BL). Seedlings were grown in the light for 7 days. Error bars represent SEM (n = 42–64). (TIF) [file pgen.1007904.s001.tif]

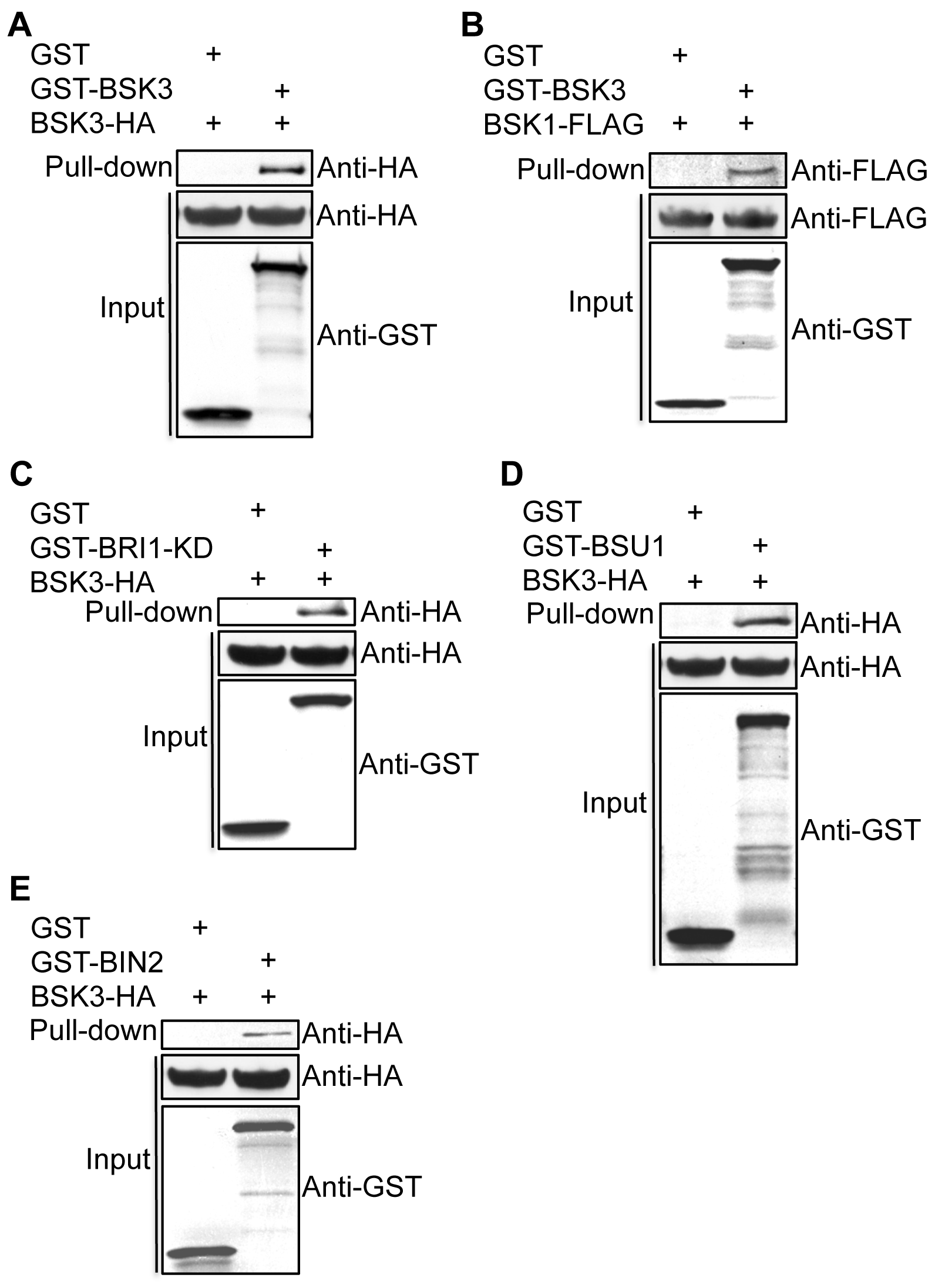

Supplement: S3 Fig — GST pull-down assays detecting BSK3-HA interactions with GST-BSK3 (A), GST-BRI1-KD (C), GST-BSU1 (D), and GST-BIN2 (E) and BSK1-FLAG and GST-BSK3 interaction (B). GST, GST fusion proteins, BSK3-HA, and BSK1-FLAG synthesized by the TNT SP6 high-yield wheat germ protein expression system were used for GST pull-down assays. (TIF) [file pgen.1007904.s003.tif]

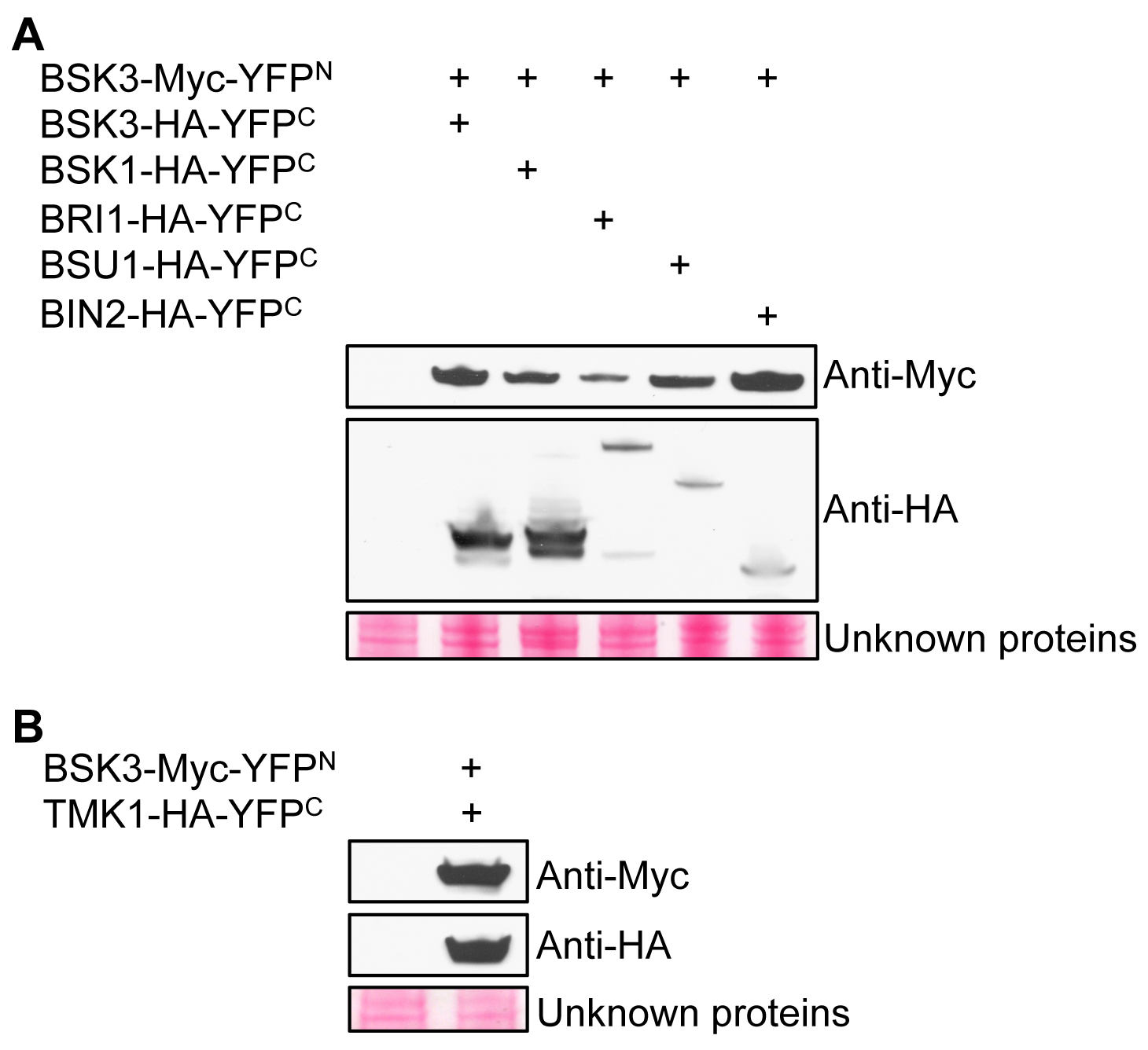

Supplement: S4 Fig — (A and B) Twenty-five micrograms of microsomal proteins were loaded. Unknown proteins stained with Ponceau S are shown as loading controls. (TIF) [file pgen.1007904.s004.tif]

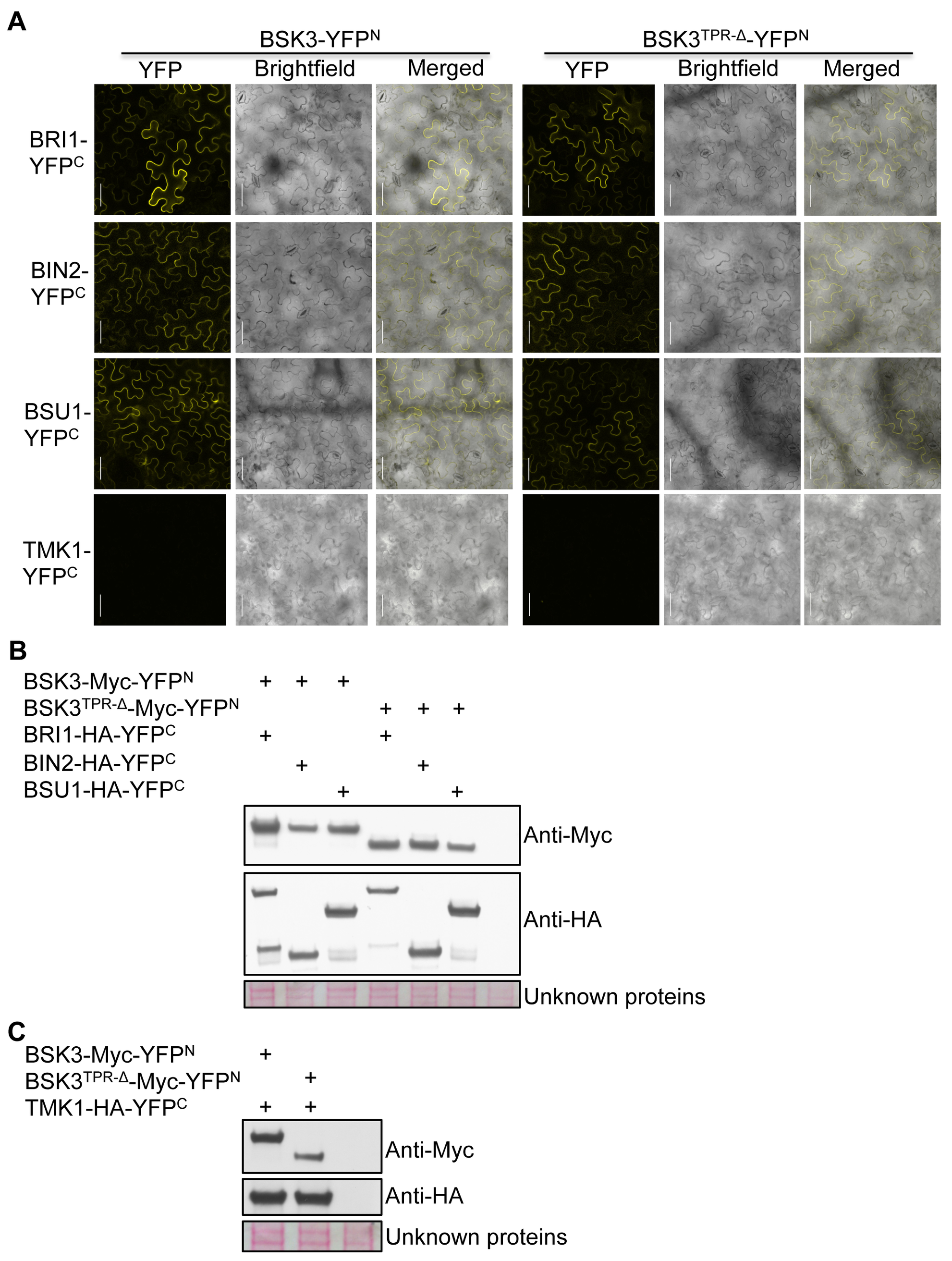

Supplement: S5 Fig — (A). BiFC assays in Nicotiana benthamiana leaves. Scale bar = 50 μm. (B and C) Western blot analyses of transiently expressed proteins in Nicotiana benthamiana leaves. Twenty-five micrograms of microsomal proteins were loaded. Unknown proteins stained with Ponceau S are shown as loading controls. (TIF) [file pgen.1007904.s005.tif]

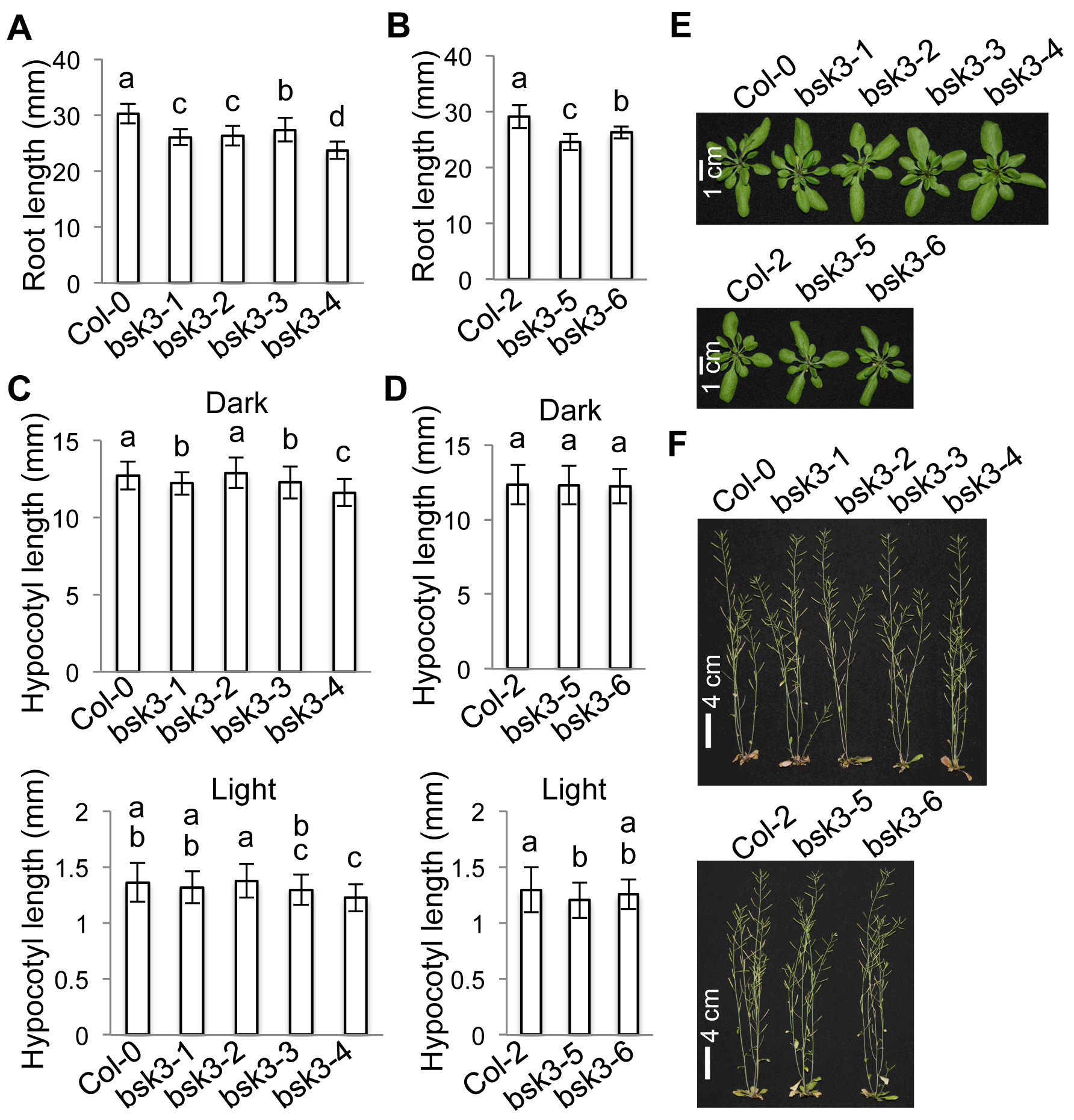

Supplement: S6 Fig — (A and B) Primary root length of 7-day-old light-grown seedlings. (C and D) Hypocotyl length of 7-day-old etiolated and light-grown seedlings. (E) Rosette leaves of 4-week-old plants. Primary bolts were removed to better observe rosette leaves. (F) Forty-eight-day-old plants. (A-D) Different letters above the bars indicate significant differences (P < 0.05). Error bars represent STD (n = 47–91). (TIF) [file pgen.1007904.s006.tif]

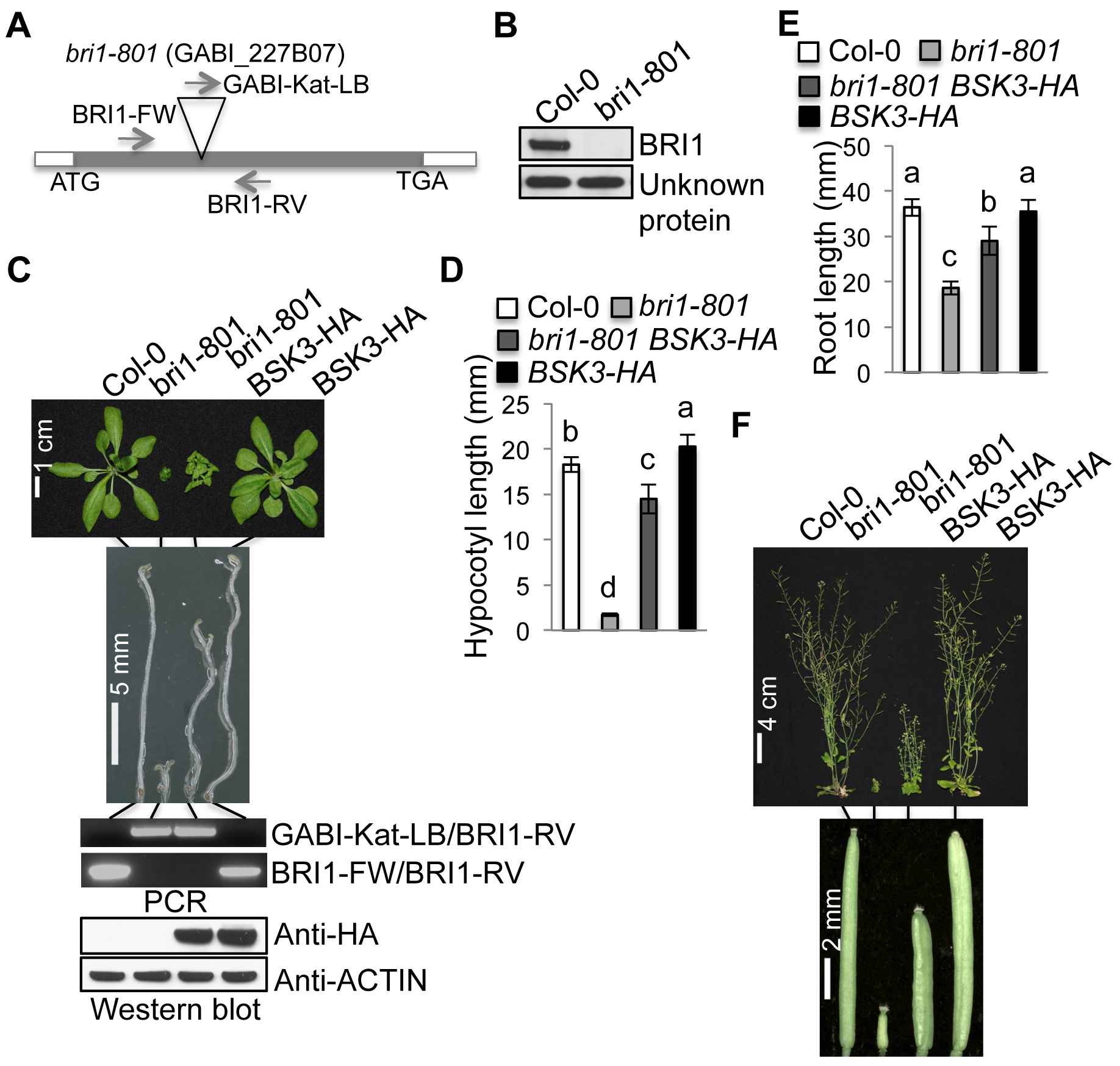

Supplement: S7 Fig — (A) The gene structure of BRI1. White boxes represent 5’ UTR and 3’ UTR, and gray box represents exon. Triangle represents the T-DNA insert, and the insertion site is between 1668 bp and 1669 bp in the exon of BRI1. (B) Western blot analysis of BRI1 protein expression. Twenty micrograms of microsomal proteins from 9-day-old light-grown seedlings were loaded. BRI1 was detected by an anti-BRI1 antibody. An unknown protein recognized by the anti-BRI1 antibody is shown as a loading control. (C) Twenty-two-day-old plants and etiolated 7-day-old seedlings. PCR primers used for genotyping bri1-801 are shown in (A). Thirty micrograms of total proteins were loaded for western blots. BSK3-HA and ACTIN proteins were detected by anti-HA and anti-ACTIN antibodies, respectively. (D) Hypocotyl length of 7-day-old etiolated seedlings. (E) Primary root length of 7-day-old light-grown seedlings. (F) Fifty-day-old plants and siliques. (D and E) Different letters above the bars indicate significant differences (P < 0.05). Error bars represent SD (n = 41–75). (TIF) [file pgen.1007904.s007.tif]
