# Supplementary material for: BRASSINOSTEROID-SIGNALING KINASE 3, a plasma membrane-associated scaffold protein involved in early brassinosteroid signaling
Source: PLoS Genet. 2019 Jan 7;15(1):e1007904. doi: 10.1371/journal.pgen.1007904 (PMC6336344; doi:10.1371/journal.pgen.1007904)
Supplement: S2 Fig — The kinase domains of the BSK family proteins were predicted by SMART. Protein sequence alignment was performed using Clustal Omega (http://www.ebi.ac.uk/Tools/msa/clustalo/). (PDF) [file pgen.1007904.s002.pdf]

```
                          BSK3K86R (kinase dead)
BSK3     EYIVSEHGEKAPNVVYKGKLE---NQKKIAVKRFTRMAWPDSRQFLEEARSVGQLRSERM 57
BSK4     ENVVSEHGETAPNVVYQGKLE---NHMKIAIKRFSGTAWPDPRQFLEEARLVGQLRSKRM 57
BSK7     -------VKKLQMLCIKGNWI---IKDVLLLKGLIGKLGLILVSFWRK-KAVGQLRNYRM 49
BSK8     ENIVSEHGERAPNVVYKGKLE---NQRRIAVKRFNRKSWPDSRQFLEEAKAVGQLRNHRM 57
BSK5     -------------VVYKGRLE---DDRWIAVKRFNRSAWPDTRQFLEEAKAVGQLRNERL 44
BSK6     DNIVSEHGEKAPNVVYRGRLD---DGRLIAVKRFNRLAWADHRQFLDEAKAVGSLRSDRL 57
BSK9     -------------IVYKGQLN---DGRKIAVKRFQRLSWPDSLEFIEEAQAVGRCRSEHM 44
BSK10    -------------IVYKGKLG---DGRRIAVKRFQRLSWPDPFEFINEAQAVGRLRSEHM 44
BSK2     -------------VVYRGKLE---GNHLVAIKRFSRQSWPDAQQFVVEATGVGKLRNKRI 44
BSK11    --------------VYQGCIRSDKDKRLIAVKKFSKTTWPDPKQFATEARAIGSLRHVRL 46
BSK1     --------------VYKGRLQ---NRRWIAVKKFTKMAWPEPKQFAEEAWGVGKLRHNRL 43
BSK12    -------------VVFKGRLQ---NGGFVAIKRFNNMAWSDPKLFLEEAQRVGKLRHKRL 44
                           :*           : :* :           *  :   :*  *  ::

                                         BSK3R156K (bsk3-3)
BSK3     ANLLGCCCEGDERLLVAEFMPNETLAKHLFHWETQPMKWTMRLRVVLYLAQALEYCTSKG 117
BSK4     ANLLGYCCEGGERLLVAEFMPNETLAKHLFHWDTEPMKWAMRLRVALYISEALEYCSNNG 117
BSK7     ANLLGCCYEGEERLLVAEFMPNETLAKHLFHWESQPMKWAMRLRVALHIAQALEYCTGKG 109
BSK8     ANLLGCCYEDEERLLIAEFMPNETLAKHLFHWESQPMKWAMRLRVALHIAQALEYCTSKG 117
BSK5     ANLIGFCCEGDERLLVAEFMPFETLSKHLFHWDSQPMKWSMRLRVALYLAQALEYCSSKG 104
BSK6     ANLIGCCFEGEERLLVAEFMPHETLAKHLFHWENNPMKWAMRLRVALCLAQALEYCSNKG 117
BSK9     ANLIGCCSEGHERLLVAEYMPNETLAKHLFHWEKRPMKWEMRLRVALHTATALEYCNDWG 104
BSK10    ANLIGCCCDDNERLLVAEYMPNGTLAKHLFHWEKRPMKWEMRLKVALHTARALEYCNDKG 104
BSK2     VSLIGCCAEGDERLLVAEYMPNDTLSKHLFHWEKQPLPWDMRVRIADYIAEALDYCNIEN 104
BSK11    VNLIGYCCEGDERLLVSEYMPNESLTKHLFHWEKQTMEWAMRLRVALYVAEALEYCRQSG 106
BSK1     ANLIGYCCDGDERLLVAEFMPNDTLAKHLFHWENQTIEWAMRLRVGYYIAEALDYCSTEG 103
BSK12    VNLIGYCCDGDKRFLVADFMANDTLAKRLFQRKYQTMDWSIRLRVAYFVAEALDYCNTAG 104
         ..*:* * :. :*:*::::*.  :*:*:**: . ..: * :*:::    : **:**   .

BSK3N182AQAL insertion (bsk3-5)             BSK3G226E (bsk3-4)
BSK3     RTLYHDLNAYRVLFDEECNPRLSTFGLMKNSRDGKSYSTNLAFTPPEYLRTGRITPESVI 177
BSK4     HTLYHDLNAYRVLFDEECNPRLSTFGLMKNSRDGKSYSTNLAFTPPEYLRTGRITAESVI 177
BSK7     RALYHDLNAYRVLFDDDSNPRLSCFGLMKNSRDGKSYSTNLAFTPPEYLRTGRVTPESVM 169
BSK8     RALYHDLNAYRVLFDDDANPRLSCFGLMKNSRDGKSYSTNLAFTPPEYLRTGRVTPESVI 177
BSK5     RALYHDLNAYRILFDQDGNPRLSCFGLMKNSRDGKSYSTNLAFTPPEYLRTGRVIPESVV 164
BSK6     RALYHDLNAYRVLFDKDGNPRLSCFGLMKNSRDGKSYSTNLAFTPPEYLRTGRVTPESVV 177
BSK9     IDLYHDLNTYRILFDKVGNPRLSCFGLMKCSREGKSYSTNLAFAPPEYLRLGTVIPESVT 164
BSK10    IDLYHDLNPYRIMFDKTGIPKLSCFGLMKNSHEGKIYSTNLAFAPPEYLRLGTVIAESVT 164
BSK2     RKIYHDLNAYRILFDEEGDPRLSTFGLMKNSRDGKSYSTNLAYTPPEFLRTGRVIPESVI 164
BSK11    LKLYHDLNTCRVLFDENGSPRLSCFGWMKNSKDGKNFSTNLAYTPPEYLRS-----ESVV 161
BSK1     RPLYHDLNAYRVLFDEDGDPRLSCFGLMKNSRDGKSYSTNLAYTPPEYLRNGRVTPESVT 163
BSK12    FASYNNLSAYKVLFDEDGDACLSCFGLMKEINNDQ-------------ITTGSVNPENVI 151
             *::*.. :::**.   . ** ** **  .:.:             :       *.*

  BSK3G238S (bsk3-6)
BSK3     YSFGTLLLDLLSGKHIPPSHALDLIRDRNLQTLTDSCLDGQFSDSDGTELVRLASRCLQY 237
BSK4     YSFGTLLLDLLTGKHIPPSHALDLIRDRNLQTLTDSCLEGQFSDSDGTELVRLTSCCLQY 237
BSK7     YSYGTLLLDLLSGKHIPPSHALDLIRDRNIQMLIDSCLEGQFSSDDGTELIRLASRCLQY 229
BSK8     YSFGTLLLDLLSGKHIPPSHALDLIRDRNIQMLMDSGLEGQFSSDDGTELIRLASRCLQY 237
BSK5     YSFGTLLLDLLSGKHIPPSHALDLIRGKNFLMLMDSCLDGHFSNDDGTDLVRLASRCLQY 224
BSK6     FSFGTVLLDLMSGKHIPPSHALDLIRGKNCAMLMDSALEGHFSNEDGTELVRLATRCLQY 237
BSK9     FSFGTLLLDLMSGRHIPPNHALDLFRGKNYLVLMDSALDGQFSDEDRTELIHLASRCLRP 224
BSK10    FSFGTLLLDLMSGRHIPPNHALDLFRGKNYLVLMDSALDGQFSDEDRTELIHVASRCFKT 224
BSK2     FSYGTILLDLLSGKHIPPSHALDIIRGKNALLLMDSSLEGQYANDDATKLVDLASKCLQS 224
BSK11    FSFGTFLLDLLSGKHIPPSHAVGTIQKQNLNVLMDSHLEGNYPEEDAAMVFDLASKCLHN 221
BSK1     YSFGTVLLDLLSGKHIPPSHALDMIRGKNIILLMDSHLEGKFSTEEATVVVELASQCLQY 223
BSK12    YRFGTVLVNLLSGKQIPPSHAPEMIHRKNVFKLMDPYLKGKFSIDEANVVYKLASQCLKY 211
         : :**.*::*::*::***.**    :: :*    * *. *.*::. .:   :  ::: *::

BSK3     EARERPNTKSLVTALTPL---- 255
BSK4     EARERPNIKSLVTAL------- 252
BSK7     EPRERPNPKSLVTAMI------ 245
BSK8     EPRERPNPKSLVSAM------- 252
BSK5     EARERPNVKSLVSSL------- 239
BSK6     EARERPNVKSLVTSL------- 252
BSK9     EPDERPSIKFLMSALSRL---- 242
BSK10    EPEERPSIKFLKATLSRLQKRA 246
BSK2     EAKDRPDTKFLLSAV------- 239
BSK11    NPNERPEIGDIISVI------- 236
BSK1     EPRERPNTKDLVATL------- 238
BSK12    EGQESPNTKEIVATL------- 226
         :  : *.   : : :
```
