# Supplementary material for: BRASSINOSTEROID-SIGNALING KINASE 3, a plasma membrane-associated scaffold protein involved in early brassinosteroid signaling
Source: PLoS Genet. 2019 Jan 7;15(1):e1007904. doi: 10.1371/journal.pgen.1007904 (PMC6336344; doi:10.1371/journal.pgen.1007904)
Supplement: S1 Table — (PDF) [file pgen.1007904.s008.pdf]

S1 Table. T-DNA insertion mutants of the *BSK* family genes.

Genes	Mutants	T-DNA insertion positions
BSK1	*bsk1-1* (GABI_180B09)	5’ UTR
BSK2	*bsk2-1* (SALK_001600)	Exon 5
BSK3	*bsk3-1* (SALK_096500C)	Intron 1
BSK4	*bsk4-1* (SALK_032845C)	Intron 6
BSK5	*bsk5-1* (SALK_074467)	Intron 7
BSK6	*bsk6-1* (SALK_063711C)	Exon 5
BSK8	*bsk8-1* (SALK_077982)	Exon 4
BSK10	*bsk10-1* (SALK_016806)	Exon 5
BSK11	*bsk11-1* (GABI_063A06)	Exon 7
BSK12/SSP	*ssp*-2 (SALK_051462)	Intron 2
