# Supplementary material for: BRASSINOSTEROID-SIGNALING KINASE 3, a plasma membrane-associated scaffold protein involved in early brassinosteroid signaling
Source: PLoS Genet. 2019 Jan 7;15(1):e1007904. doi: 10.1371/journal.pgen.1007904 (PMC6336344; doi:10.1371/journal.pgen.1007904)
Supplement: S2 Table — (PDF) [file pgen.1007904.s009.pdf]

**S2 Table. PCR primers used in this study.**

| Primers | Sequences (5' to 3' direction) |
|---|---|
| **RT-PCR primers** | |
| ACTIN2-FW | GGCTGAGGCTGATGATATTC |
| ACTIN2-RV | TCTGTGAACGATTCCTGGAC |
| BSK3-HA-FW | CTTTAGAGAGGCCATCGAGTG |
| HA-RV | CGTAATCTGGAACATCGTATGG |
| BSU1-EYFP-FW | GAAAGATTCGCTGACGGACGGC |
| EYFP-RV | CAGCTTGCCGTAGGTGGCATCG |
| BSK3-FW | GATGGGAGGTCAATGCTCTAGCC |
| BSK3-RV | ACTGTCGTGAATCAGGCCAGG |
| **Mutant genotyping primers** | |
| BSK1-GABI_180B09-FW | CTACTCTATTTAGAAGGCACGTC |
| BSK2-SALK_001600-FW | GGGATATGCGTGTTCGAATCGC |
| BSK3-SALK_096500C-FW | GAGTCTAACTAGTCGAGTTGAC |
| BSK4-SALK_032845C-RV | AGATGTCAGGCGTACTAACTCC |
| BSK5-SALK_074467-RV | GTTCATGTACATACCTCATTTGC |
| BSK6-SALK_063711C-RV | GGTCAAGCGCCTGTATAGAAGC |
| BSK8-SALK_077982-RV | TGAAACCTTAGCTGTCAGCTGG |
| BSK10-SALK_016806-RV | CGGCATCTCCATGCTTCTTG |
| BSK11-GABI_063A06-FW | CAAACATTTCGAATCTGCTATTGA |
| BSK12-SALK_051462-RV | GAAGAGGCACAAAGAGTAGGG |
| BRI1-FW | CTCCGGTCCGATTCTCCCAAATC |

| | |
|---|---|
| BRI1-RV | GAACCAGAGATATCGTTATGACCC |
| SALK-LBb1.3 | ATTTTGCCGATTTCGGAAC |
| GABI-Kat-LB | ATAATAACGCTGCGGACATCTACATTTT |
| bin2-1-FW | CACGATTCTACCGTGCACCCGAG |
| bin2-1-RV | GACCAGTTCAGGTGATGATCCAGC |
| **Site-directed mutagenesis primers** | |
| $BSK3^{K86R}$-FW | CCAAAAGAAGATTGCTGTCAGGCGTTTTACTAGAATGGC |
| $BSK3^{K86R}$-RV | GCCATTCTAGTAAAACGCCTGACAGCAATCTTCTTTTGG |
| $BSK3^{R156K}$-FW | CCTATGAAATGGACTATGAAGCTACGAGTTGTTTTATATC |
| $BSK3^{R156K}$-RV | GATATAAAACAACTCGTAGCTTCATAGTCCATTTCATAGG |
| $BSK3^{G226E}$-FW | GAATATCTACGAACTGAGAGAATTACTCCAGAG |
| $BSK3^{G226E}$-RV | CTCTGGAGTAATTCTCTCAGTTCGTAGATATTC |
| $BSK3^{G238S}$-FW | GTGTAATATACAGCTTTAGCACTCTTCTGCTTGATC |
| $BSK3^{G238S}$-RV | GATCAAGCAGAAGAGTGCTAAAGCTGTATATTACAC |
| $BRI1^{K911E}$-FW | GCGCGGTGGCTATCGAGAAACTGATTCATG |
| $BRI1^{K911E}$-RV | CATGAATCAGTTTCTCGATAGCCACCGCGC |
| $BIN2^{K69R}$-FW | GAAACCGTGGCGATAAGGAAGGTTTTGCAAG |
| $BIN2^{K69R}$-RV | CTTGCAAAACCTTCCTTATCGCCACGGTTTC |
